# Interconnected lineage trajectories link conventional and natural killer (NK)-like exhausted CD8^+^ T cells beneficial in type 1 diabetes

**DOI:** 10.1038/s42003-024-06456-3

**Published:** 2024-06-27

**Authors:** Erin M. Witkop, Kirsten Diggins, Alice Wiedeman, Elisavet Serti, Gerald Nepom, Vivian H. Gersuk, Bryce Fuchs, S. Alice Long, Peter S. Linsley

**Affiliations:** 1 Benaroya Research Institute, Systems Immunology, Seattle, WA USA; 2 Benaroya Research Institute, Translational Immunology, Seattle, WA USA; 3 Immune Tolerance Network (ITN), Bethesda, MD USA; 4 Benaroya Research Institute, Genomics Core, Seattle, WA USA

**Keywords:** Type 1 diabetes, Immunosuppression, Immunological memory

## Abstract

Distinct Natural Killer (NK)-like CD57^+^ and PD-1^+^ CD8^+^ exhausted-like T cell populations (Tex) have both been linked to beneficial immunotherapy response in autoimmune type 1 diabetes (T1D) patients. The origins and relationships between these cell types are poorly understood. Here we show that while PD-1^+^ and CD57^+^ Tex populations are epigenetically similar, CD57^+^ Tex cells display unique increased chromatin accessibility of inhibitory Killer Cell Immunoglobulin-like Receptor (iKIR) and other NK cell genes. PD-1^+^ and CD57^+^ Tex also show reciprocal expression of Inhibitory Receptors (IRs) and iKIRs accompanied by chromatin accessibility of Tcf1 and Tbet transcription factor target sites, respectively. CD57^+^ Tex show unappreciated gene expression heterogeneity and share clonal relationships with PD-1^+^ Tex, with these cells differentiating along four interconnected lineage trajectories: Tex-PD-1^+^, Tex-CD57^+^, Tex-Branching, and Tex-Fluid. Our findings demonstrate new relationships between Tex-like populations in human autoimmune disease and suggest that modulating common precursor populations may enhance response to autoimmune disease treatment.

## Abstract (web summary)

RNA-sequencing data from NK-like CD57^+^ and PD-1^+^ CD8^+^ exhausted-like T cell populations linked to beneficial immunotherapy response in autoimmune patients suggest shared clonal relationships with each other and with common precursor populations.

## Introduction

T cell exhaustion is driven by prolonged antigen exposure and is characterized by increased expression of cell surface Inhibitory Receptors (IRs) (e.g., PD-1, LAG3, CTLA4, and TIGIT) and diminished effector function^1–3^. In cancer patients, T cell exhaustion may result in failure to eliminate tumors, which can be therapeutically reversed by reinvigorating exhausted-like T cells (Tex) cells using inhibitory receptor blockade (e.g., anti-PD-1)^2,4^. In autoimmune disease, increased CD8^+^ T cell exhaustion may be associated with slower disease progression^5–7^.

Tex form a heterogeneous cell lineage with distinct populations marked by key epigenetic and transcriptomic signatures^3,8,9^. In patients with autoimmune type 1 diabetes (T1D), increased Tex population levels were previously linked to improved outcomes following two T cell-targeted therapies^10–12^. Specifically, improved response to alefacept (LFA3-Ig) in patients with recent onset T1D was associated with changing levels of two expanded and hypoproliferative Tex populations which co-expressed TIGIT and KLRG1 and displayed an EOMES gene signature^10,12^. These TIGIT^+^KLRG1^+^ (double positive, DP) Tex populations were distinguished by high PD-1 expression (PD-1^+^ Tex) or CD57 and natural killer (NK) cell-associated gene expression (CD57^+^ Tex), including inhibitory killer cell immunoglobulin-like receptor (iKIR) genes^12^. NK cell-like CD8^+^ T cell populations have also been identified in human patients with MS and mice with lymphocytic choriomeningitis virus infection (LCMV)^9,13^.

These studies support the potential therapeutic benefits of inducing or modulating Tex cells in autoimmunity generally, and T1D specifically^7,14^. However, the role of distinct Tex populations in T1D outcome and what cues determine their differentiation remain largely unknown, hindering efforts to design treatments that precisely augment beneficial Tex subsets.

Here, we used epigenetic and transcriptomic profiling to investigate lineage relationships of PD-1^+^ and CD57^+^ Tex populations. We specifically compared the chromatin accessibility, single-cell gene expression, and TCR profiles of PD-1^+^ Tex, CD57^+^ Tex, and TIGIT^-^KLRG1^-^ nonnaïve double negative (DN) cells. Our results characterize PD-1^+^ and CD57^+^ Tex populations associated with beneficial responses to immunotherapy and demonstrate interconnected lineage trajectories between PD-1^+^ and CD57^+^ Tex populations, suggesting potential targets for future therapeutic intervention.

## Results

### CD8^+^ PD-1^+^ and CD57^+^ Tex populations are stable and distinct, yet share key chromatin accessibility features

**Fig. 1:**
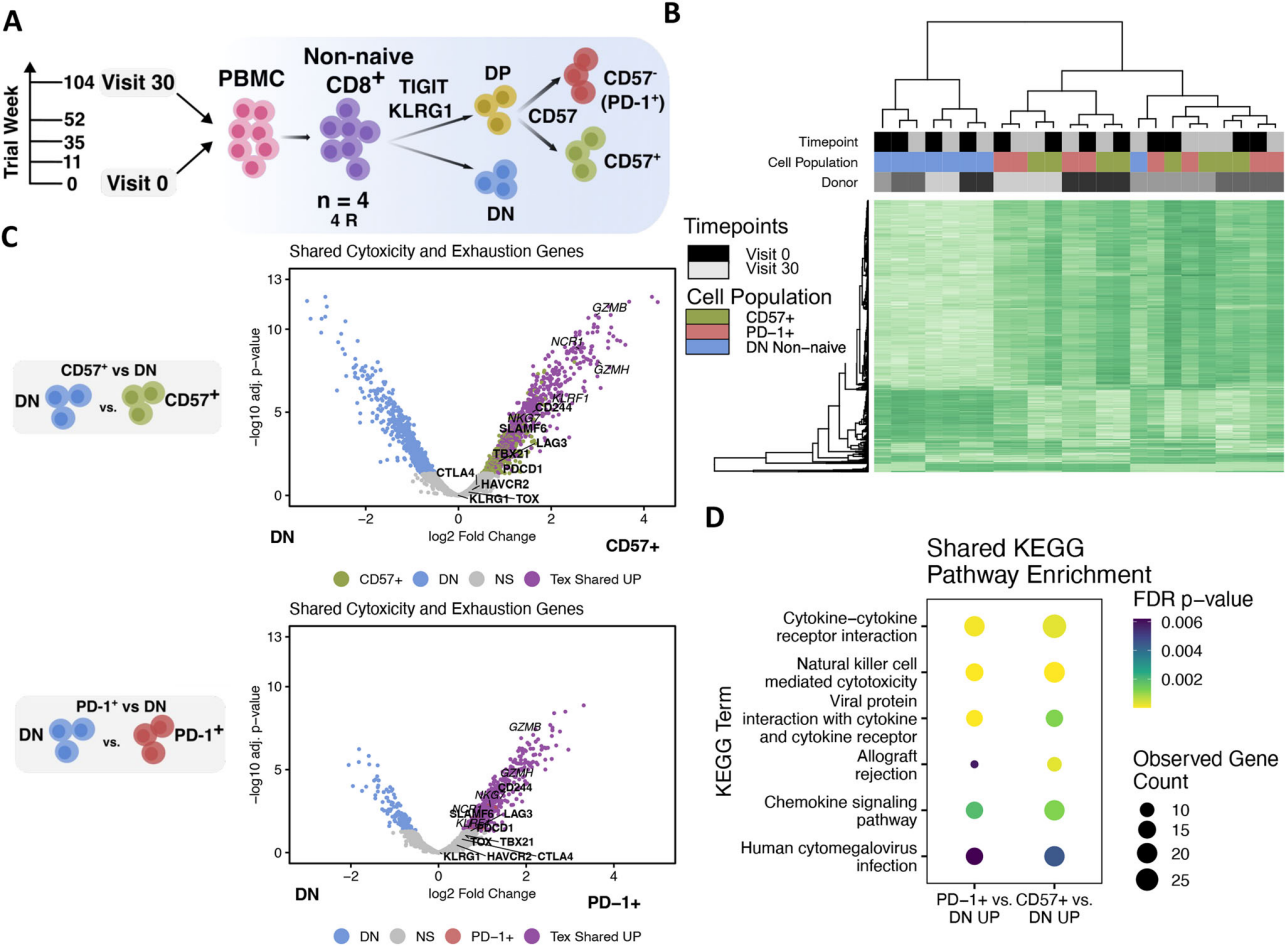
Stable and distinct CD8^+^ PD-1^+^ and CD57^+^ Tex populations share increased chromatin accessibility of exhaustion and cytotoxicity genes. **A** Schematic depicting the number of alefacept-treated donors and timepoints that were sampled for ATAC-seq (*n* = 4 R, R, Visit 0 = baseline, Visit 30 = 104 wk post-treatment) and the cell sorting scheme used to isolate nonnaïve CD8^+^ T cell populations into either TIGIT^-^KLRG1^-^ double negative (DN), or TIGIT^+^KLRG1^+^ (DP) CD57^+^ Tex or PD-1^+^ Tex (24 samples total; PD-1^+^ Tex sorted as TIGIT^+^KLRG1^+^ CD57^-^ population based on previous work^10^, see methods). **B** Heatmap clustering of all consensus sites per sample (columns). Color blocks indicate sample cell population, donor, and timepoint. **C** Significance (-log10 FDR-adjusted *p* value) and log2 fold change of all differentially accessible peaks (FDR *p* value ≤ 0.05) between CD57^+^ Tex vs DN and PD-1^+^ Tex vs DN. Key genes related to exhaustion (bold) and cytotoxicity (italicized) are labeled. Where multiple peaks were annotated to the same gene, only the peak with the greatest log2 fold change was plotted. Shared genes across comparisons are highlighted in purple. NS not significant. Labeled genes that are below the cutoffs are still regulated in the same direction (i.e., the same side of the volcano), but their regulation is weaker. **D** Observed gene count and FDR-adjusted *p* value of KEGG pathways with shared enrichment in both CD57^+^ Tex and PD-1^+^ Tex populations versus DN samples.

To characterize the exhaustion profiles and lineage relationships of the TIGIT^+^KLRG1^+^ PD-1^+^ and TIGIT^+^KLRG1^+^ CD57^+^ Tex populations over time, both of which were previously shown to be hypoproliferative and to express exhaustion markers^12^, we first measured their bulk chromatin accessibility at baseline (Week 0/Visit 0) and 104 wk (Visit 30) post-treatment in alefacept responders (R, defined by C-peptide preservation) using ATAC-seq^15^ following sorting from PBMC (Fig. 1A, Supplementary Data 1, and Supplementary Fig. 1)^16^. Because cell population differences rather than treatment response differences were the focus of our investigation, we sequenced only alefacept responder (R) samples. To first characterize general epigenetic similarity between samples, we clustered the full open chromatin region (OCR) signatures of all samples in a heatmap (Fig. 1B). PD-1^+^ and CD57^+^ Tex clusters were intermixed with one another, regardless of donor and timepoint, with all but one DN sample clustering together. Additionally, no peaks were differentially accessible (DA) between timepoints in any cell population (FDR-adjusted *p* value ≤ 0.05). These data show that PD-1^+^ and CD57^+^ Tex populations were epigenetically similar and stable between timepoints.

Since chromatin changes in T cell exhaustion are largely stable and irreversible and Tex show distinct stages over time^3,8,17^, we hypothesized that greater sharing of differentially accessible sites would support lineage sharing between populations. To determine if populations were epigenetically distinct but from a shared lineage, we compared changes between PD-1^+^ Tex or CD57^+^ Tex versus the TIGIT^-^KLGR1 DN population (Fig. 1C and Supplementary Data 1). We used DN cells as a reference population because we previously found they were less exhausted than TIGIT^+^KLGR1^+^ cells^10^. Both PD-1^+^ and CD57^+^ Tex populations had many more OCRs than the DN population (CD57^+^ Tex vs DN = 5821 DA OCRs; PD-1^+^ Tex vs DN = 3530 DA OCRs). Many OCRs had increased DA in both PD-1^+^ and CD57^+^ Tex populations vs DN (2837 “Tex Shared Up”), representing 65 and 94% of increased DA sites in CD57^+^ and PD-1^+^ Tex populations, respectively (Supplementary Fig. 2), supporting a common lineage between these populations. However, the presence of many additional unique sites in the CD57^+^ Tex population suggests that these populations were, at least in part, epigenetically distinct.

To better understand shared functionality between PD-1^+^ and CD57^+^ Tex populations, we assessed KEGG pathway gene enrichment in shared and increased DA genes of both Tex populations compared to DN (Fig. 1D). Terms for the KEGG pathways NK cell-mediated cytotoxicity (hsa04650, *p* value = 3.90e-06) and cytokine–cytokine receptor interaction (hsa04060, *p* value = 1.20e-04) were significantly enriched among shared genes, suggesting the importance of these pathways in the potential functions of both populations. Additionally, both populations shared increased chromatin accessibility of several T cell exhaustion and cytotoxicity markers^13,18^ including *GZMB*, *GZMH*, *CD244*, *NKG7*, *PRF1, and PDCD1* (Fig. 1C), and the NK-activating receptors *NCR1* and *KLRF1*, but not NK inhibitory receptors (IR). The transcription factor *TOX* is a key marker of exhausted cells, but in our experiments, was not noted as differentially accessible. Upon closer examination, we found that *TOX* had accessible chromatin near the transcription start site in PD-1^+^ and CD57^+^ Tex populations as well as the DN reference cells (Supplementary Fig. 3). Thus, both PD-1^+^ and CD57^+^ Tex populations were characterized by open chromatin near the 5’end of the *TOX* gene, as expected for exhausted cells, but at levels similar to DN cells. *TOX* gene open chromatin in the DN reference cells likely reflects that these are effector-like cells^12^ which also express *TOX*^4^. Chromatin accessibility was also increased near *LAG3*, and *TBX21* (T-bet) genes in both PD-1^+^ and CD57^+^ Tex cells, but these differences were only significant in the CD57^+^ Tex population. Both of these genes have been associated with exhausted cells^3,19^, although they may have other roles as well^20^. Overall, these results support the shared exhausted nature of both PD-1^+^ and CD57^+^ Tex populations, but differences in chromatin accessibility between populations supported additional heterogeneity.

### Increased Inhibitory KIR accessibility distinguishes CD8^+^ TIGIT^+^KLRG1^+^ CD57^+^ Tex population

**Fig. 2:**
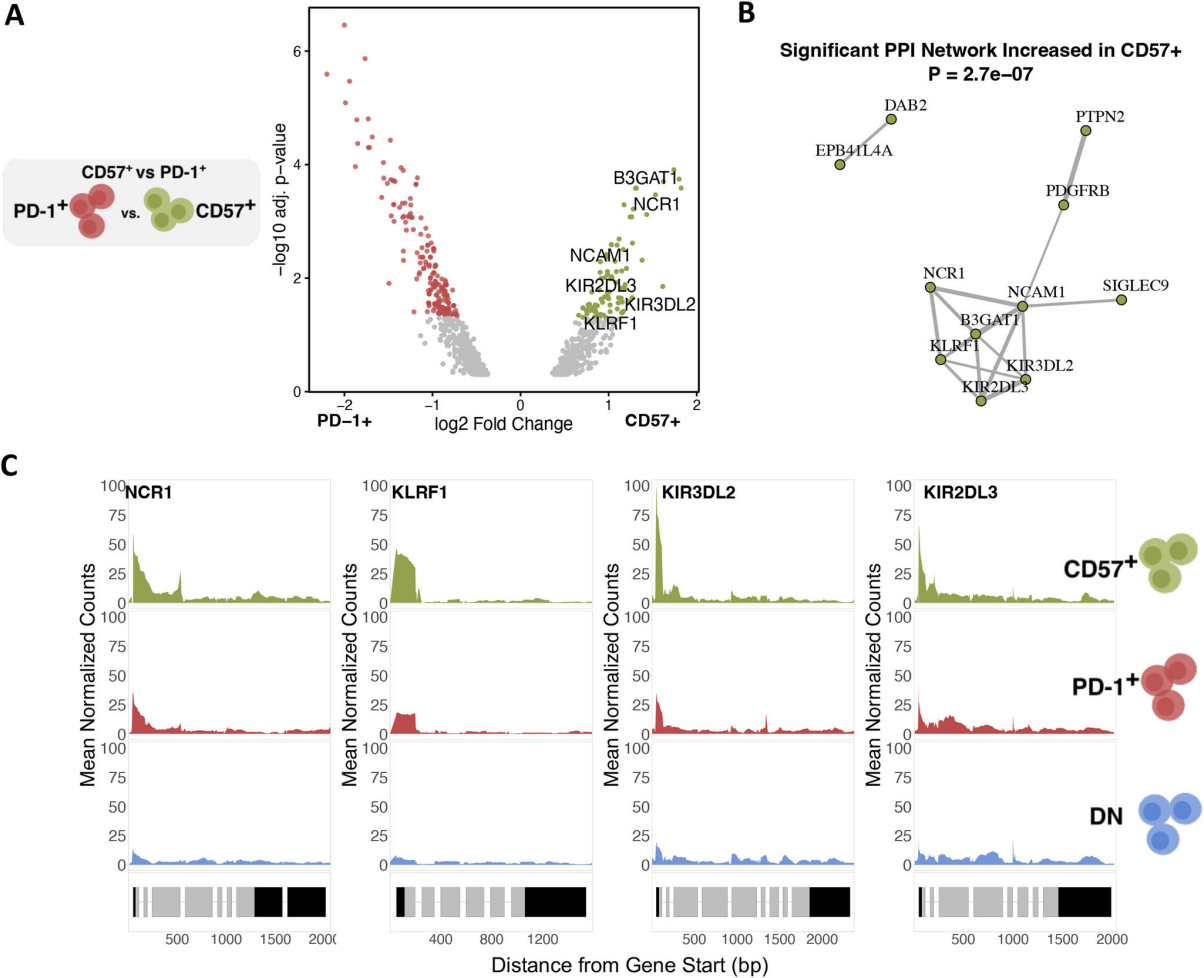
Inhibitory KIR genes have increased chromatin accessibility in the CD8^+^ TIGIT^+^KLRG1^+^ CD57^+^ Tex population. **A** Significance (-log10 FDR-adjusted p-value) and log2 fold change of differentially accessible sites between CD57^+^ Tex vs PD-1^+^ Tex populations sorted from CD8^+^ nonnaïve memory cells (*n* = 4 R, sampled at baseline and 104 wk post-treatment). NK-like genes expressed in the CD57^+^ Tex population are labeled. **B** Enriched protein–protein interaction network in differentially accessible genes with increased accessibility in the CD57^+^ Tex population. Edges are weighted by the strength of protein–protein interactions. The PPI enrichment *p* value was calculated using *STRING*. **C** Mean ATAC peak coverage for key NKRs and iKIRs with increased accessibility in CD57^+^ vs PD-1^+^ Tex populations shown for all three sample groups.

We next compared PD-1^+^ and CD57^+^ Tex chromatin accessibility to differentiate their functions (Supplementary Data 1). The PD-1^+^ Tex population had 195 annotated OCRs with increased accessibility compared to CD57^+^ Tex (Fig. 2A). These genes formed a significant protein–protein interaction network (FDR-adjusted *p* value = 4.4e-05) but were not enriched in immune-related pathway terms (Supplementary Fig. 4). Fewer (*n* = 97) OCRs had increased accessibility in the CD57^+^ Tex population compared to the PD-1^+^ Tex population, but they were more uniformly NK-related genes. These included NK-activating receptors *NCR1* and *KLRF1* and iKIRs *KIR3DL2*, and *KIR2DL3* (Fig. 2A, C). Genes with increased accessibility in the CD57^+^ Tex population formed a significant protein–protein interaction network (FDR-adjusted *p* value = 2.7e-07) where *KIR3DL2* and *KIR2DL3*, along with *KLRF1*, *NCR1*, *NCAM1*, and *B3GAT1* gene involved in *CD57* biosynthesis^21^) were central network constituents (Fig. 2B). To demonstrate the robustness of increased NK-related gene accessibility, we compared mean ATAC peak counts for these NK cell receptors (NKRs) across CD57^+^ Tex, PD-1^+^ Tex, and DN populations and showed they were higher in CD57^+^ Tex for all four NKR OCRs (Fig. 2C). These results support iKIRs as key pathway constituents that differentiate CD57^+^ Tex from PD-1^+^ Tex.

### Heterogeneity in CD57^+^ Tex population and reciprocal inhibitory receptor expression in Tex populations revealed by scRNA-seq

**Fig. 3:**
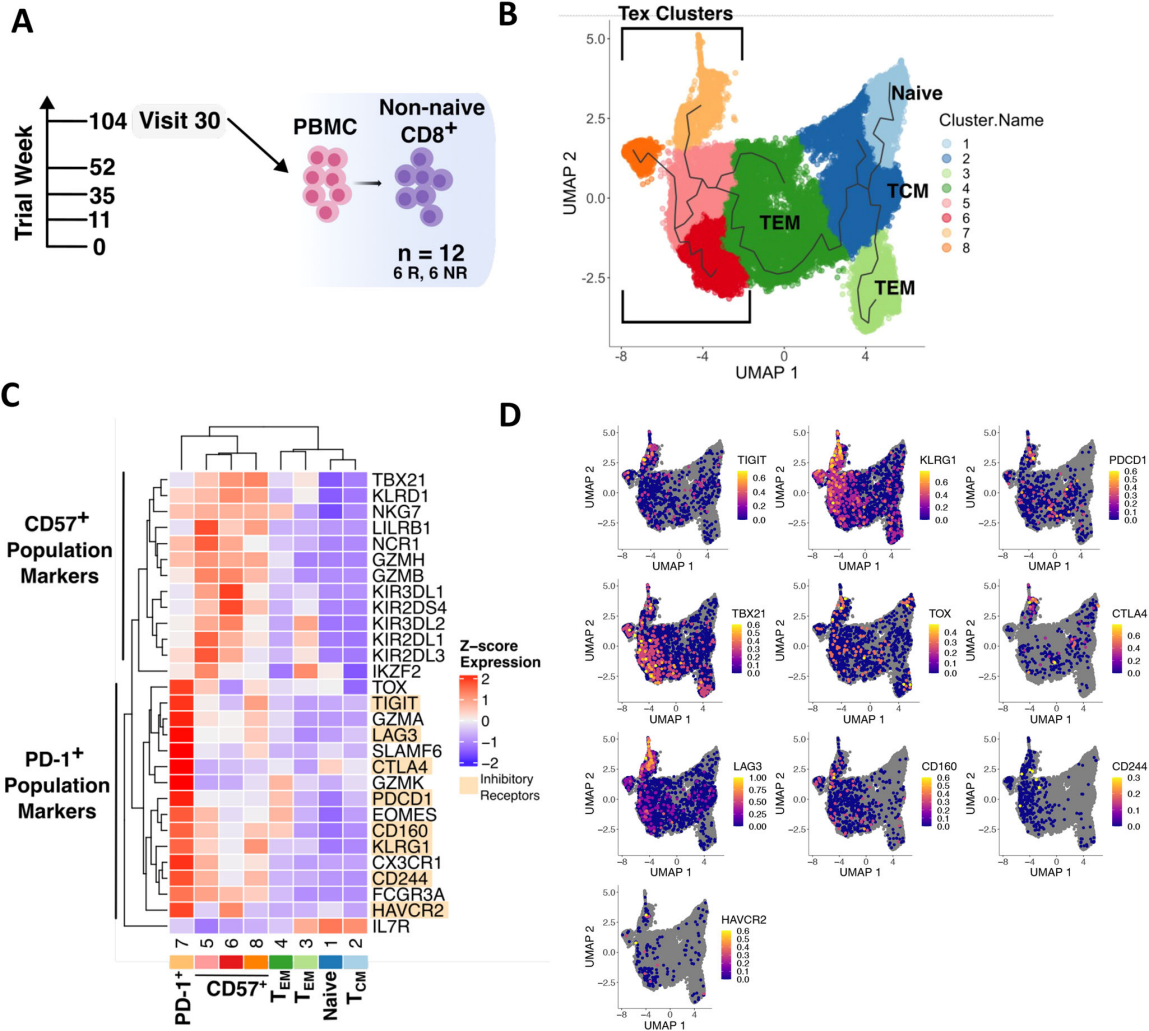
TIGIT^+^KLRG1^+^ CD57^+^ Tex population has increased single-cell gene expression heterogeneity and reciprocal expression of Inhibitory Receptors. **A** Schematic of scRNA-seq sampling strategy; *n* = 12 donors, 1 sample per donor (6 R, 6 NR, sampled at Visit 30, 104 wk post-treatment). **B** UMAP dimensionality reduction of all cells clustered using the Leiden clustering method, with branching lines depicting the cell trajectory determined by Monocle. Phenotype assignment labels were based on Seurat reference mapping of CD8^+^ T cell phenotypes and expression of Tex markers (Supplementary Figs. 6c,  8)^10^. **C** Z-score adjusted mean expression in each cluster of known key markers of exhaustion and PD-1^+^ and CD57^+^ Tex populations, where Inhibitory Receptors (IRs, orange highlight) displayed a reciprocal expression pattern between PD-1^+^ and CD57^+^ Tex populations. **D** Log10 normalized single-cell gene expression of key exhaustion-related genes. More differentiated cells on the left of the UMAP dimensionality reduction display higher exhaustion-related gene expression.

While PD-1^+^ and CD57^+^ Tex populations shared epigenetic patterns of exhaustion and cytotoxicity, suggesting a shared lineage, epigenetic and transcriptome states do not always mirror each other^17^. To further explore heterogeneity in Tex populations, we compared the transcript diversity of PD-1^+^ and CD57^+^ Tex populations to chromatin accessibility signatures by performing scRNA-sequencing on CD8^+^ nonnaïve memory cells sorted from PBMC from 6 R and 6 Non-responders (NR), at 104 wk post-treatment (*n* = 12 total samples, Supplementary Fig. 5 and Fig. 3A). We clustered the full gene expression profiles of all 26,978 cells using the Leiden clustering method and visualized them in two dimensions using uniform manifold approximation and projection (UMAP) dimensionality reduction. This identified eight distinct groups, or clusters, of cells that were mixed evenly between R and NR groups (Fig. 3b and Supplementary Figs. 6a, b). CD8^+^ reference mapping identified less differentiated or more terminally differentiated and exhausted-like cells on opposite sides of the UMAP dimensionality reduction (Supplementary Fig. 6c). The percentage of cells in each Tex cluster was not correlated with C-peptide slope, a key measure of response to alefacept in this trial (Supplementary Fig. 7a, b)^22^. We associated PD-1^+^ and CD57^+^ Tex populations with different clusters by assessing their similarity to CD8^+^ reference populations^23^ and previously identified PD-1^+^ and CD57^+^ Tex gene signatures^12^. Heatmap clustering of PD-1^+^ and CD57^+^ Tex markers^12^ showed *PDCD1* was most expressed in cluster 7 and strongly clustered with IR expression (Fig. 3C). CD57^+^ Tex population markers, including *NCR1*, *LILRB1*, *KLRD1*, *GZMB*, *NKG7*, and KIRs, were most expressed in clusters 5, 6 and 8 (Fig. 3C). These results supported identification of cluster 7 as the PD-1^+^ Tex population and clusters 5, 6, and 8 as CD57^+^ Tex population clusters. Importantly, IRs and KIR genes were reciprocally expressed between PD-1^+^ and CD57^+^ Tex clusters, with IRs *CTLA4*, *TIGIT*, *LAG3*, *HAVCR2*, and *PDCD1* more strongly expressed in the PD-1^+^ Tex cluster and KIR genes more strongly expressed in CD57^+^ Tex clusters (Fig. 3D and Supplementary Fig. 8a-b). Together, the expression patterns of multiple markers associated with exhausted CD8^+^ T cells were consistent with the exhausted-like nature of both PD-1^+^ Tex and CD57^+^ Tex cells (Figs. 1A,  3D and Supplementary Fig. 8a, b).

We identified three unique CD57^+^ Tex clusters (clusters 5, 6, 8), demonstrating gene expression heterogeneity in this cell population (Fig. 3C and Supplementary Data 2)^12^. The top specifically expressed genes in clusters 5 and 6 were similar (*FGFBP2, GZMB, GZMH, and NKG7; GZMB, C20orf24, FGFBP2,* and *GNLY*, respectively) (Supplementary Fig. 9), revealing overlap in the top markers for these clusters. More markers were specifically expressed in cluster 8, including genes involved in the Type 1 Interferon pathway (*IFIT1, IFIT2, IFIT3, OASL, HERC5, ISG20,* and *Z3HAV1*) and signaling pathway genes (*TNF, CCL5, CCL4, CCL3L1,* and *CCL4L2*). KIR expression was also heterogenous among CD57^+^ Tex clusters (Fig. 3C and Supplementary Fig. 8). Top markers for TEM clusters 3 and 4 showed elevated levels of genes involved in immune activation (*FOS* and *JUN*) (Supplementary Fig. 9).

**Fig. 4:**
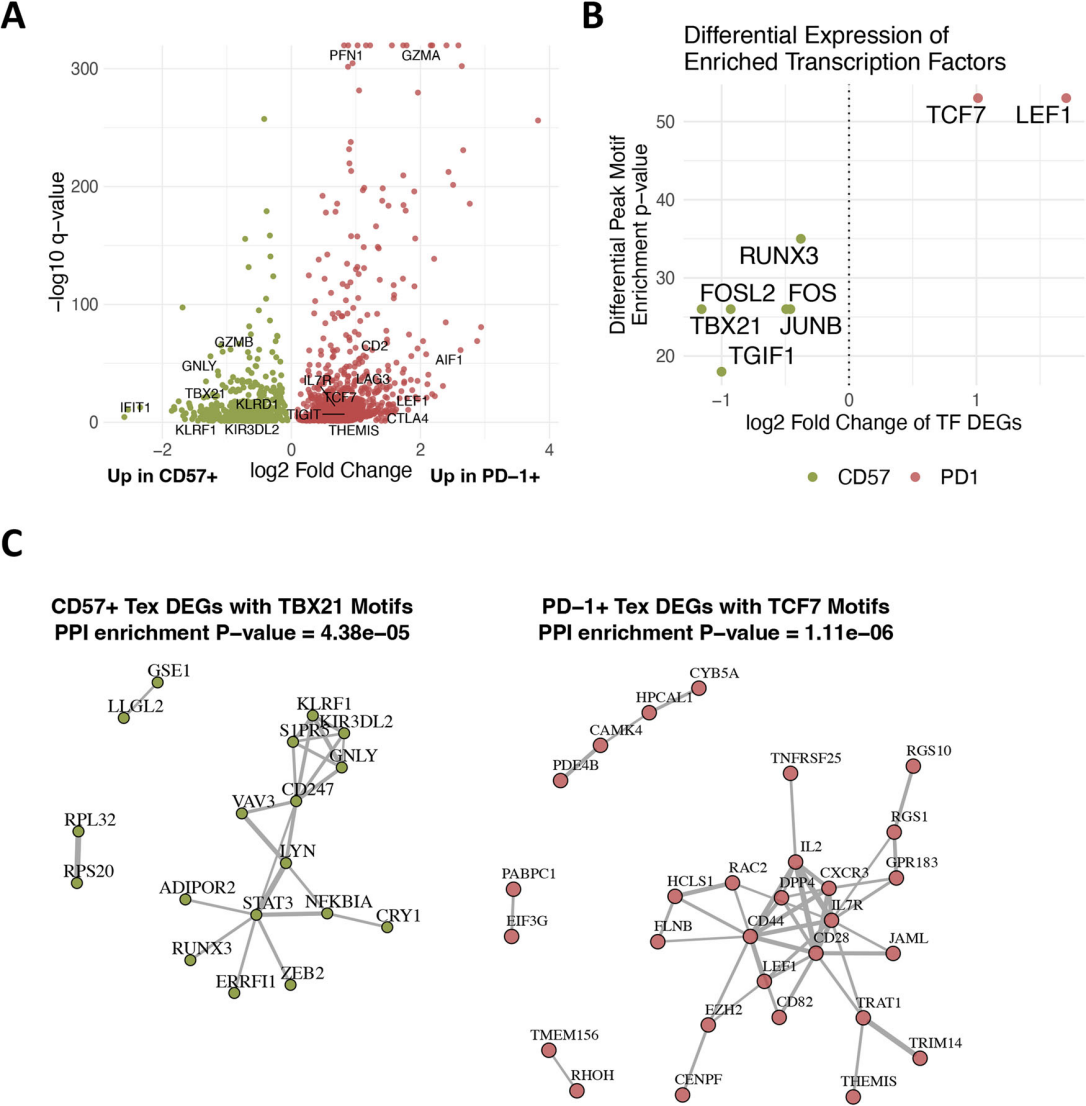
T-bet and Tcf-1 have differentially accessible binding motifs and are differentially expressed between CD57^+^ and PD-1^+^ Tex populations. **A** Significance (-log10 FDR-adjusted *p* value) and log2 fold change of differentially expressed genes between the PD-1^+^ Tex population cluster (cluster 7) and all CD57^+^ Tex population clusters (clusters 5, 6, 8) (*n* = 12, 6 R, 6 NR, sampled at 104 wk post-treatment). **B** Significance of transcription factor binding motif enrichment among differentially accessible peaks (y-axis) (*n* = 4 R, sampled at baseline and 104 wk post-treatment) versus the normalized log2 fold change of these TFs when their single-cell gene expression was compared between CD57^+^ and PD-1^+^ Tex scRNA-seq clusters (x-axis) (*n* = 12, 6 R, 6 NR, 104 wk post-treatment). **C** Enriched protein–protein interaction networks of differentially expressed genes in the CD57^+^ Tex population (left) that contained TBX21 motifs, or differentially expressed genes in the PD-1^+^ Tex population (right) that contained significant TCF7 motifs. Edges were weighted by the combined score of the protein–protein interaction and only DEGs with TF motif enrichment *p* value ≤ 0.001 were plotted. The PPI enrichment *p* values were calculated using *STRING*.

Since the CD57^+^ Tex population was distinguished from the PD-1^+^ Tex population by increased NKR chromatin accessibility, we determined whether CD57^+^ Tex clusters also had significantly greater expression of NKR and other genes. We identified differentially expressed genes (DEGs) and pathways between PD-1^+^ and CD57^+^ Tex clusters and compared these with DA genes between populations. About 753 genes were differentially upregulated in CD57^+^ Tex clusters versus 908 differentially upregulated genes in the PD-1^+^ Tex cluster (Fig. 4A and Supplementary Data 3). DEGs in CD57^+^ Tex clusters included interferon pathway genes (*IFIT1*, *IFIT2*, *IFIT3*, *IFNGR1*, *TBK1*, *OASL*) and genes involved in NK-cell-mediated cytotoxicity (*KLRD1*, *KIR3DL2*, *GNLY*, *GZMB, KLRF1*)^9,13^ Only four DEGs overlapped with differentially accessible genes but included NKRs *KIR3DL2* and *KLRF1*. In the PD-1^+^ Tex population, cytoskeleton-related genes were most upregulated and only 12 PD-1^+^ Tex DEGs overlapped with genes with increased accessibility versus the CD57^+^ Tex population, including IR genes *LAG3* and *PDCD1*. Though differentially expressed and differentially accessible genes overlapped little between populations, patterns of key NKRs *KIR3DL2* and *KLRF1* in the CD57^+^ Tex population and IRs in the PD-1^+^ Tex population were conserved.

### Differential gene expression between alefacept R and NR subjects

It was important to explore how gene expression variation between R and NR subjects contributed to gene expression differences between PD-1^+^ and CD57^+^ Tex populations. A total of 635 genes were differentially expressed between R and NR in CD57^+^ Tex population clusters (Supplementary Data 4 and Supplementary Fig. 10a), including *KIR3DL2*, *KIR2DL3*, *KLRC1*, and *KLRB1*. Fewer genes were differentially expressed between R vs NR within the PD-1^+^ Tex population (204 DEGs, Supplementary Fig. 10b). A minority of these genes (264 in the CD57^+^ Tex population) were also differentially expressed between PD-1^+^ and CD57^+^ populations (Supplementary Fig. 10c). Thus, a large fraction of gene expression differences between PD-1^+^ and CD57^+^ Tex populations were linked to drug responsiveness.

CD57^+^ Tex cells have not been universally reported in other studies of Tex cells. To place these cells into a fuller context, we compared CD57^+^ Tex cells to other CD8^+^ T cell populations. We first compared gene expression in CD57^+^ Tex cells with other published gene signatures. The KIR^+^ CD8^+^ versus KIR^-^ CD8^+^ gene expression signature from ref. ^13^ separately clustered PD-1^+^ and CD57^+^ Tex cell signatures (Supplementary Fig. 11a). This signature contained genes preferentially expressed in both clusters but not in other CD8^+^ T cell populations. Likewise, the Tex^KLR^ versus Tex^Term^ CD8^+^ T cell signature from ref. ^9^, though comprising different genes than the Li et al. signature, drove the same cell clustering pattern (Supplementary Fig. 11b). We also assessed the similarity of CD57^+^ Tex cells with other NK-like, KIR-expressing, gene sets. CD57^+^ Tex DEGs (Supplementary Data 6) were significantly enriched for the ref. ^13^ and ref. ^9^ gene sets, as well as canonical cancer Tex gene sets^24^; and Tex-KLR gene sets in mice^9,18^ (Supplementary Fig. 11c, d). The gene set most enriched was the exhaustion-associated EOMES gene module we used previously to identify Tex cells linked to response to another biologic agent, teplizumab^10^. *EOMES* may be associated with exhaustion^25^, but in other contexts cooperates with TBX21 to coordinate cytotoxic effector cells^26^. *EOMES* is also important in memory T cells^27^. Yet another NK-like CD8 + T cell population is the NKG2C^+^ population associated with prior human cytomegalovirus (HCMV) exposure^28^. We note that these cells differ from our CD57^+^ cells in several important ways, in that CD57^+^ Tex cells, unlike *NKG2C* (*KLRC2*) cells^28^: did not differentially express *NKG2C* (*KLRC2*) (Supplementary Data 3); did show expression of inhibitory receptors, such as *TIGIT*, *KLRG1*, *PDCD1*, and *LAG3*, albeit at lower levels than PDCD1^+^ Tex (Fig. 3D); and did not proliferate during CD3/CD28 stimulation in vitro^12^.

Multiple studies, although beset by technical issues, have shown *CD57* and *KLRG1* in terminally differentiated memory T cells are among the best markers available to describe an immunosenescent state^29^. Since both markers were found on CD57+ Tex cells, it was of interest to investigate whether these cells were senescent. We therefore tested genes over-expressed by CD57^+^ Tex clusters 5, 6, and 8 and PD-1^+^ Tex cluster 7, for enrichment of multiple senescence gene sets^30–33^. For comparison, we also tested multiple Tex and NK gene sets^34^. We found that none of the senescence gene sets were strongly enriched in PD-1^+^ or CD57^+^ Tex-specific genes (Supplementary Fig. 12). Senescence genes were more significantly enriched (to varying extents, depending on the senescence gene set) in some of the Tex and NK-related gene sets (termTexSet, gzmkTexSet, and nkTxSet). These findings provided no evidence that CD57^+^ Tex cells were senescent, despite their overexpression of *KLRG1* and *CD57*.

It was of interest to determine whether PD-1+ and CD57^+^ Tex cells were found in islet or viral antigen-specific T cells and if the presence of these cells predicts better response to therapy. Low frequencies of islet and viral antigen-reactive CD8^+^ T cells were found in peripheral blood of the alefacept-treated subjects. These antigen-reactive cells contained both PD-1^+^ and CD57^+^ Tex cells at levels that did not vary significantly between R and NR subjects (Supplementary Fig. 13a).

Finally, since CD57^+^ Tex cells share features of terminally differentiated cells, it was important to distinguish them from CD45RA^+^ terminally differentiated effector memory cells (TEMRA) cells. Flow cytometry showed that the parental populations of PD-1+ and CD57+ Tex cells were partially TEMRA^+^, with both populations having elevated levels relative to DN cells (Supplementary Fig. 13b). CD57^+^ Tex cells had higher levels of TEMRA cells, but still contained ~40% effector memory (EM) cells. The fraction of TEMRA cells in PD-1^+^ and CD57^+^ Tex populations differed slightly at baseline and did not change over time in the study after drug treatment (Supplementary Fig. 13c). To assess how the percentage of TEMRA cells in the CD57^+^ Tex population affected our ATAC-seq analysis, we calculated a “CD57^+^ Tex ATAC Score” (Methods). We then plotted the CD57^+^ Tex ATAC Score versus the percentage of TEMRA cells as measured by flow cytometry in the cell populations that were sorted from each donor for ATAC-seq analysis (Supplementary Fig. 13d). The Score for each donor did not vary by the percentage of TEMRA cells. Finally, we compared epigenetic marks that distinguish CD57^+^ Tex from PD-1^+^ cells with a series of differentially accessible ATAC-seq signatures of TEMRA cells from Giles et al.^35^ (Supplementary Fig. 13e). The most relevant signature tested, of peaks differing between TEMRA and PD-1^+^CD39^+^ exhausted cells, did not show significant overlap with the CD57^+^ Tex cell signature. This contrasts with the strong overlap between the Giles et al. exhausted gene set and CD57^+^ Tex cells (Supplementary Fig. 11c). The only TEMRA signature that showed significant overlap was a TEMRA versus effector memory cell (EM) signature, perhaps indicating shared differences between TEMRA and CD57^+^ Tex cell with EM cells. Taken together, these data indicate that while CD57^+^ Tex cells contain a substantial fraction of TEMRA cells, they are not identical to TEMRA cells. The CD57^+^ Tex cell signatures likely reflect the exhausted-like nature of these cells rather than simply a terminally differentiated phenotype.

### T-bet and Tcf-1 may be regulators of CD57^+^ and PD-1^+^ Tex population differentiation

Transcription factors (TF) are known to drive differentiation pathways of Tex^3^. To explore potential TFs that regulate CD57^+^ and PD-1^+^ Tex differential expression, we identified enriched TF motifs within differentially accessible chromatin regions using HOMER^36^ (Supplementary Data 5) and compared these enriched motifs with differentially expressed genes between populations. Binding motifs for *TBX21* (T-bet), *RUNX3*, and *FOS* were highly enriched among DARs in CD57^+^ Tex compared to the PD-1^+^ Tex population, and the expression of these TFs was also increased in CD57^+^ Tex vs PD-1^+^ Tex. (Fig. 4B). *TCF7* (Tcf-1) and *LEF1* TF motifs were highly enriched among DARs in the PD-1^+^ Tex populations and TCF7 was differentially expressed in PD-1^+^ Tex vs the CD57^+^ Tex population. To assess whether these TFs may play a role in regulating differential gene expression, we examined whether key DEGs near regions of open chromatin (as defined by GREAT^37^) contained *TBX21* and *TCF7* binding motifs (Fig. 4C). In the CD57^+^ Tex population, *TBX21* motifs were identified in or near multiple DEGs, including *KIR3DL2* and *KLRF1* genes, and in the PD-1^+^ Tex population, *TCF7* motifs were identified in or near the differentially expressed genes including *CD28*, *LEF1*, and *IL7R* (shown in a network graph in Fig. 4C). These data suggest a potential differential role for T-bet and Tcf-1 as regulators of the CD57^+^ and PD-1^+^ Tex populations, respectively.

### PD-1^+^ and CD57^+^ Tex populations are clonally expanded and share a common DN progenitor

**Fig. 5:**
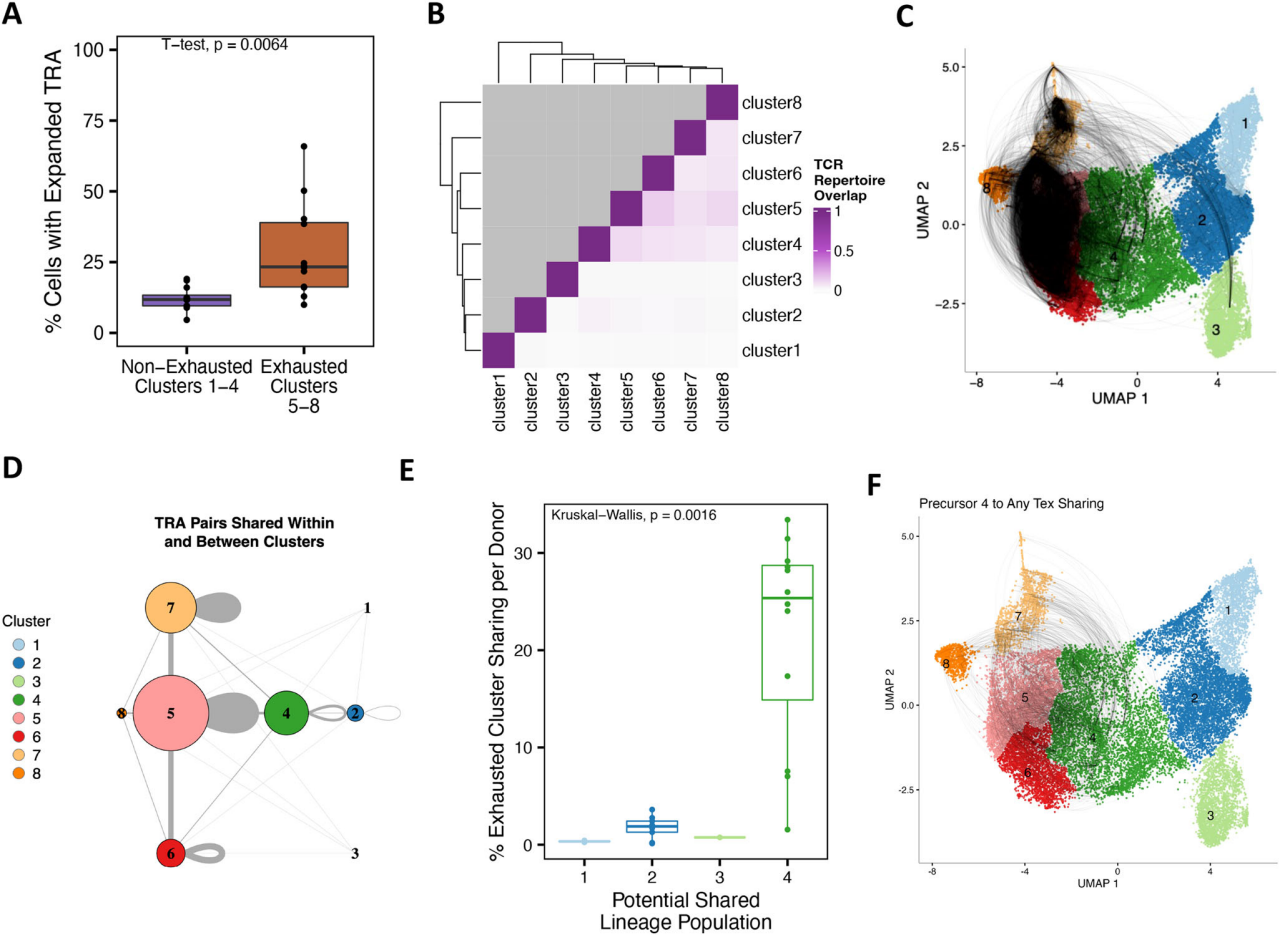
TCR sharing and cell trajectory reveal that CD57^+^ and PD-1^+^ Tex populations are expanded and share a common lineage. **A** We compared the percentage of cells with an expanded *TRA* (>1 cell with *TRA*) per donor in either Tex clusters (clusters 5, 6, 7, 8) or non-exhausted clusters (clusters 1, 2, 3, 4) (all donors sampled for combined scRNA-seq and TCR-seq; *n* = 12, 6 R, 6 NR, 104 wk post-treatment). Population differences were determined using a Student’s *t*-test. **B** The similarity of unique *TRA* repertoires between combinations of clusters was measured using the Jaccard similarity coefficient (0, no *TRA* chain sharing between clusters; 1, all *TRA* chains shared) and visualized using a heatmap of clustered Jaccard similarity values. Duplicates of the bottom half of the matrix are grayed out. **C** UMAP dimensionality reduction connecting cells with a shared *TRA* chain by a curved line. **D** Summary of *TRA* sharing in a network visualization (*TRB* shown in Supplementary Fig. 15a), with custom layout and color scheme replicating scRNA-seq Leiden clustering and UMAP dimensionality reduction layout and color^51^. Nodes (circles) are sized by the total number of cells with a sequenced TCR per cluster. Straight edges (gray lines) indicate *TRA* sharing between clusters. Curved edges (gray loops) indicate *TRA* expansion within a cluster. The edge width is scaled to represent the level of *TRA* sharing. **E** percentage of Tex cells (clusters 5, 6, 7, 8) per donor that shared a *TRA* chain with each potential precursor cluster (1, 2, 3, or 4). Boxes extend from the first to the third quartiles (interquartile range) with a line in the middle that represents the median. Lines extending from the boxes (whiskers) represent variability outside the interquartile range. Points represent individual donors. Population differences were determined using a Kruskal–Wallis test. **F** UMAP dimensionality reduction connecting cells that share a *TRA* chain between potential precursor population 4 and each Tex cluster (5, 6, 7, or 8) by a curved line.

While CD57^+^ and PD-1^+^ Tex populations have key epigenetic and gene expression differences, as well as similarities suggesting a shared lineage, their precise lineage relationships remain unknown. We coupled TCR sequencing with the same samples sequenced for scRNA-seq^38^ (CD8^+^ nonnaïve memory cells, *n* = 12, 6 R, 6 NR, 104 wk post-treatment) to determine whether PD-1^+^ and CD57^+^ Tex populations were expanded, whether populations shared a common lineage, and if populations had divergent or shared differentiation trajectories. TCR recovery varied by donor (Supplementary Fig. 14a) and by UMAP cluster (Supplementary Fig. 14b, c). While both *TRA* and *TRB* chains considered individually gave very similar results, for simplicity, only data regarding *TRA* chains are presented in the main figures. To investigate whether Tex populations were more expanded than non-exhausted populations, we compared the percentage of clonally expanded cells (*TRA* sequence in >1 cell per cluster) in exhausted-like PD-1^+^ and CD57^+^ Tex clusters (clusters 5–8) versus non-exhausted clusters (clusters 1–4). A significantly greater percentage of cells in exhausted-like cell clusters had expanded *TRA* chains than non-exhausted clusters (*t*-test, *p* value = 0.0064), supporting the expansion of Tex populations (Fig. 5A).

To determine whether PD-1^+^ and CD57^+^ Tex populations shared a common progenitor and lineage, we utilized TCR chains as barcodes to link clonally related cells across all samples (CD8^+^ nonnaïve memory cells, *n* = 12, 6 R, 6 NR, 104 wk post-treatment). We calculated how many unique *TRA* chains were shared between clusters and used this value to calculate Jaccard similarity coefficients as a metric of similarity of two sets. Clustering these values in a heatmap (Fig. 5B) showed clusters 4–8 had the highest and most similar Jaccard similarity coefficients, and Jaccard similarity did not differ between CD57^+^ or PD-1^+^ Tex populations.

We also visualized patterns of clonal expansion by connecting cells with a shared *TRA* chain with an arc connecting their UMAP dimensionality reduction coordinates (Fig. 5C). These “airline” plots revealed many connections within and between exhausted-like clusters, often originating with cluster 4. To summarize these patterns, we plotted the total number of shared connections across the UMAP dimensionality reduction as a network (Fig. 5D and Supplementary Fig. 15a). This indicated between-cluster sharing was greatest between clusters 4, 5, 6, and 7 and showed few clones were shared from clusters 1, 2, or 3 with any exhausted-like cluster (5–8). The clusters that shared the most *TRA* chains differed between individual donors when visualized as a heatmap (Supplementary Fig. 15b), though patterns of sharing in R and NR donors did not cluster together. The percentage of CD57^+^ Tex and PD-1+ Tex cells per donor that share a TCR is higher in cluster 4 than in other potential precursor clusters, clusters 2 and 3 (Supplementary Fig. 16a). We additionally compared the percentage of exhausted-like cells (clusters 5–8) that shared a *TRA* chain with each potential progenitor cluster (cluster 1, 2, 3, or 4) (Fig. 5e). Cluster 4 and the clusters of exhausted-like cells shared the most *TRA* chains (Kruskal–Wallis, *p* value = 0.0016). Finally, we also visualized these patterns on UMAP airline plots (Fig. 5F and Supplementary Fig. 16b). These results support a shared lineage for CD57^+^ and PD-1^+^ Tex populations and suggested CD8^+^ TEM cluster 4 as a potential common progenitor population.

### Patterns of TCR sharing reveal interconnected differentiation trajectories and suggest the potential for Tex population fluidity

**Fig. 6:**
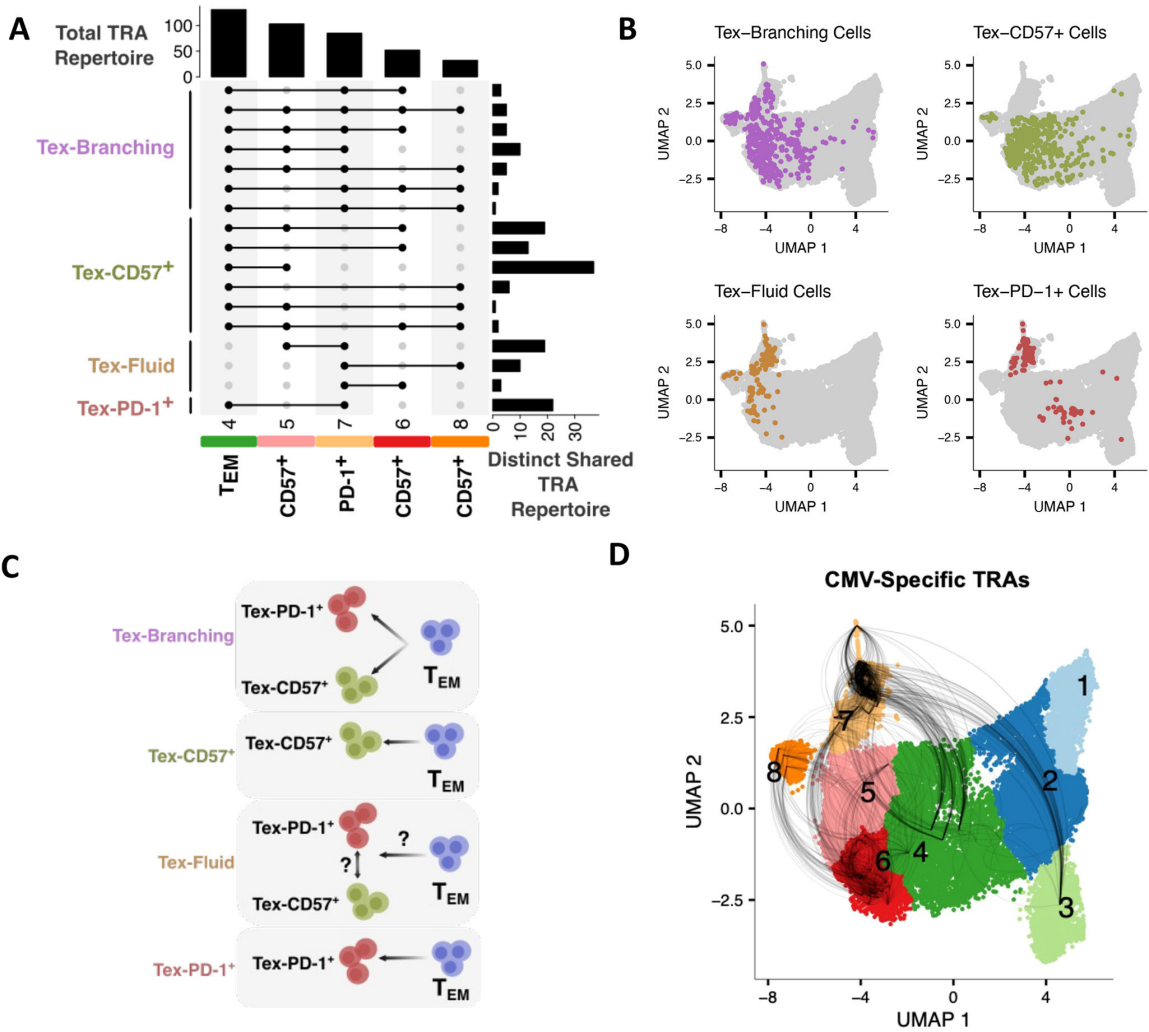
TIGIT^+^KLRG1^+^ CD8^+^ CD57^+^ and PD-1^+^ Tex populations have multiple interconnected differentiation trajectories indicating population fluidity. **A** Unique *TRA* chain sharing between combinations of clusters determined by Leiden clustering followed by UMAP dimensionality reduction (all donors sampled for combined scRNA-seq and TCR-seq; *n* = 12, 6 R, 6 NR, 104 wk post-treatment). Vertical columns show the total number of unique *TRA* chains in that cluster. Solid circles across each row outline show which *TRA* repertoires for a given set of clusters are being compared. Circles are connected by lines to enable easier viewing. Adjacent horizontal bars depict the number of unique *TRA* chains only shared between that group of clusters (e.g., The top row depicts *TRA* chains only shared between clusters 4, 7, and 6). Patterns of unique *TRA* chain sharing between cluster combinations were divided into four differentiation trajectories: Tex-Branching, Tex-CD57^+^, Tex-Fluid, and Tex-PD-1^+^. **B** UMAP dimensionality reduction highlighting cells with *TRA* chains in each differentiation trajectory. **C** Cartoon depicting cell differentiation hypotheses for each trajectory. **D** UMAP dimensionality reduction depicting cell sharing of CMV-specific *TRA*s. Cells with shared CMV specificity are connected by a curved black line.

TCR sequence analysis revealed that CD57^+^ and PD-1^+^ Tex populations were expanded and likely shared nonnaïve cluster 4 as a common progenitor population. To further explore lineage trajectories of CD57^+^ and PD-1^+^ Tex populations, we analyzed unique combinations of *TRA* chain sharing between clusters and used patterns of sharing to infer differentiation (all donors sampled for combined scRNA-seq and TCR-seq; *n* = 12, 6 R, 6 NR, 104 wk post-treatment) (Fig. 6A). We summarized *TRA* chain sharing patterns into four distinct and interconnected trajectories (Tex-PD-1^+^, Tex-CD57^+^, Tex-Branching, Tex-Fluid) based on whether each group of *TRA* chains was shared between the common progenitor cluster 4 and one or both Tex populations. Tex-PD-1^+^ cells shared *TRA* chains between cluster 4 and cluster 7 (PD-1^+^ Tex population) only, Tex-CD57^+^ cells shared *TRA* chains between cluster 4 and clusters 5, 6 or 8 (CD57^+^ Tex population clusters), Tex-Branching cells shared *TRA* chains between cluster 4 and clusters from both populations, and Tex-Fluid cells shared *TRA* chains between PD-1^+^ and CD57^+^ Tex clusters (5–7, 6–7, 8–7) and *not* cluster 4 (Fig. 6B). These trajectories suggest CD8^+^ TEM cluster 4 is a bifurcation point between Tex-PD-1^+^, Tex-CD57^+^, and Tex Branching trajectories (Fig. 6C). We were unable to identify the potential precursor population of Tex-Fluid cells, suggesting these cells may be the result of fluidity between CD57^+^ and PD-1^+^ Tex populations following differentiation or originate from a non-sampled progenitor population.

To determine whether these distinct trajectories demonstrated general biological phenomena across donors and specificities, we first compared *TRA* diversity per donor between each trajectory (all donors sampled for combined scRNA-seq and TCR-seq; *n* = 12, 6 R, 6 NR, 104 wk post-treatment). Shannon-Wiener diversity, a measure of TCR richness and evenness, did not differ between trajectories, though donors varied within groups and the Tex-Branching trajectory was the most abundant (Supplementary Fig. 17a, b). Multiple donors were also present in each trajectory.

We then investigated whether these trajectories were present in cells with known TCR antigen-specificity for common infectious agents, Cytomegalovirus (CMV) or Epstein-Barr virus (EBV)^39^, using TCR sequences found in the VDJdb repository^40^. We identified cells in our combined scRNA-seq and TCR-seq data with CMV-specific *TRA* chains in seven donors, across all differentiation trajectories, and with multiple trajectories within individual donors (Fig. 6D and Supplementary Fig. 18a, b). EBV-specific cells, however, were only present in four donors and were only identified in one trajectory, Tex-PD-1^+^. We identified and phenotyped CD8^+^ T cells specific for either CMV or EBV using cytometry by time-of-flight (CyTOF) with tetramer staining, and found they were present in either the CD57^+^ Tex or PD-1^+^ population, or both (Supplementary Fig. 18c). These results suggest that Tex cells are not restrained to a single specificity, and that a single specificity can be present in multiple Tex populations. Thus, each trajectory had similar repertoire diversity, was present across multiple donors in both R and NR groups, and was identified in both sequenced and stained tetramer-specific cells with a common antigen specificity. These findings argue that the identified trajectories may represent general biological phenomena rather than features unique to certain donors or antigen specificities.

## Discussion

CD8^+^ T cells differentiate into Tex populations that maintain stable epigenetic identities over time^3,9,17,18,41^. While relationships and features of Tex have been extensively compared with memory and effector T cells, there is not a single, universally accepted definition of Tex cells, which has led to the concept of a continuum of Tex cell states^4^. We showed previously that PD-1^+^ and CD57+ Tex showed shared characteristics widely associated with Tex, namely reduced proliferation following stimulation, and overexpression of IR^12^. Here we show that PD-1^+^ and CD57^+^ populations show additional gene expression properties characteristic of different Tex cell states, as well as altered expression and open chromatin at the transcriptional start site of the transcription factor, *TOX*. We also show that PD-1^+^ and CD57^+^ Tex populations share characteristics with each other, including many OCRs and clonal relationships as determined by TCR clonotypes. Taken together, these findings argue that CD57^+^ and PD-1^+^ cells are clonally related populations that both lie on the continuum of Tex-like cell states.

Despite their similarities, there are some differences in properties between CD57+ Tex and those reported for Tex cells. One difference is in the expression of *KLRG1* and *CD57* by CD57^+^ Tex. Some have argued that these genes are markers of senescent rather than exhausted cells^1,25^. Moreover, *KLRG1* is also highly associated with effector, rather than exhausted CD8^+^ T cells^1^. Our data in young adult HC and T1D individuals show clearly that *KLRG1* is more highly expressed in exhausted-like cells than in TEM or TEMRA cells (Fig. 3D). In addition, CD57^+^ Tex were not enriched for senescence-associated genes (Supplementary Fig. 12), nor were they enriched for TEMRA-associated ATAC-seq peaks (Supplementary Fig. 13), arguing that they did not share features of these better-known cell types. Another difference between CD57^+^ Tex and other Tex cell populations is their relatively low expression of some IRs. Careful examination shows that while CD57^+^ Tex do express lower levels of several IRs (*TIGIT, LAG3, CTLA4, CD160, PDCD1*, and *KLRG1*) than do PD-1^+^ Tex, these levels are higher than in TEM, TCM, and naive CD8 + T cells (Fig. 3C, D). None of these markers are completely specific for Tex, but all share a graded pattern of expression from naïve to more terminal cells (Fig. 3D). In addition, CD57^+^ Tex overexpress another class of IRs, the KIRs. Overall, CD57^+^ Tex are more similar than different from Tex populations, supporting the conclusion that they lie on a continuum of Tex-like cell states.

The stability of the exhaustion program in both CD57^+^ and PD-1^+^ Tex populations, regardless of treatment, supports epigenetic markers as potential identifiers of exhaustion for future therapeutic intervention. In cancer patients, epigenetic stability limits the durability of Tex reinvigoration by PD-1 blockade^7,42^. In contrast, the stability of exhaustion we observed may be leveraged in autoimmunity to drive durable effects for therapy. The CD57^+^ Tex population displayed greater gene expression heterogeneity than the PD-1^+^ Tex population by scRNA-seq analysis. This heterogeneity could be influenced by environmental cues like TCR signal strength and avidity, leading to more varied CD57^+^ Tex expression phenotypes^9,43^. The action of transcription factors T-bet (*TBX21*) and Tcf-1 (*TCF7*), whose expression and accessibility of binding motifs differed between the two Tex populations, may also influence gene expression heterogeneity. Tcf-1 is a known marker of self-renewing progenitor-like Tex populations with PD-1^+^ surface expression, while T-bet is associated with intermediate effector Tex populations^3,18,44–46^. These results highlight transcription factors and pathways that could be targeted to selectively drive differentiation of one or multiple Tex populations associated with improved immunotherapy response in T1D patients^12^.

Our study showed that iKIR chromatin accessibility and gene expression are hallmarks of the CD57^+^ Tex population. Several recent studies implicate CD8^+^ CD57^+^ Tex-like populations in control of autoimmunity and chronic infection. Regulatory KIR^+^ CD8^+^ T cells in patients with autoimmune disease and SARS-CoV-2 infection shared a strong NK cell signature with our CD57^+^ Tex population^13^, as did Killer Cell Lectin-like Receptor (KLR)-expressing Tex cells (Tex^KLR^) in mouse LCMV infection^9,25^. KIR^+^ CD8^+^ T cells can suppress self-reactivity through direct killing of pathogenic CD4^+^ T cells, while Tex^KLR^ cells are hypothesized to preserve immune homeostasis while controlling pathogen replication^13,47^. Reciprocal expression between KIR and IR genes in the CD57^+^ and PD-1^+^ Tex populations, respectively, was also observed in healthy blood^48^ and between ligands for KIR and IR genes in cancer cells^49,50^. Targeting KIR ligands in cancer can reinvigorate CD57^+^ T cells, suggesting the KIR pathway is functionally involved in suppressing immune responses^49^. Alefacept-treated patients showing slower T1D progression (R) preserved higher CD57^+^ Tex levels following treatment than patients showing more rapid disease progression, consistent with a role for CD57^+^ Tex in suppression of autoimmunity.

Using patterns of TCR sharing, we demonstrated that a CD8^+^ NK-like CD57^+^ Tex state linked to beneficial response in T1D shares a common precursor and terminal interconnections with beneficial conventional PD-1^+^ Tex states, a novel finding in human autoimmunity. Recent studies identified differentiation trajectories among CD8^+^ Tex populations in response to LCMV in mice, including Divergent, Tex^term^-biased, and Tex^KLR^-biased trajectories, and a subset of Tex^KLR^ cells that may experience fluidity between populations^9^. Mice, however, do not express KIR genes or the CD57 antigen, differentiating the linkages defined between their NK-like Tex and conventional PD-1^+^ Tex state from our study.

Our study used multiple sequencing approaches to provide significant insight into the lineage and characteristics of two Tex populations previously linked to beneficial response to alefacept treatment in T1D^51^. Small sample sizes limited our ability to explore how antigen specificity and gene expression associated with alefacept response, though we did not observe any significant differences between the percentage of islet and virus antigen-specific cells between R and NR patients (Supplementary Fig. 13a), and our ATAC-seq data suggest that treatment did not induce lasting epigenetic changes (Fig. 1B). Pairing scRNA-seq and single cell ATAC-seq in the same individual cells may aid future efforts to interrogate relationships between Tex populations. Finally, though our analysis suggested that iKIR genes and NK pathways are important for the function of CD57^+^ Tex cells, our studies were not designed to probe this relationship. Thus, how these pathways activate or inhibit the function of heterogeneous CD57^+^ Tex cells and cellular targets of these iKIRs remains unknown.

CD8^+^ T cell exhaustion has been previously linked to response to teplizumab^10,11^, which was recently approved by the FDA for prevention of T1D^52^. Thus, the present studies with alefacept represent the second example of the linkage between CD8^+^ T cell exhaustion and beneficial response to a T cell-depleting biological therapy in T1D. Future research to understand signals driving Tex differentiation and function in T1D may help design improved T cell-targeted therapies.

## Methods

### Subjects and samples

Patient samples from the T1DAL trial were collected at participating sites under the auspices of trial NCT00965458^16^. The use of patient samples at BRI was approved by the BRI Institutional Review Board (IRB07109-482). Samples from trial participants were collected from consented subjects and blinded prior to providing to researchers at BRI. Researchers received no patient identifiers and samples were unblinded only after completion of cytometry or RNA-seq analyses and submission of raw data to ITN *TrialShare* (https://www.immunetolerance.org/researchers/trialshare). All ethical regulations relevant to human research participants were followed.

### Cytometry acquisition and analyses

For flow cytometry, cells from thawed PBMC were stained (Supplementary Data 7) and sorted on a BD *FACSAria™ Fusion* Flow Cytometer using *Diva* software for downstream RNA-seq and ATAC-seq processing and analysis. Purity of sorts was determined by comparing sorted cells with negative fractions; all sorted cell populations were >90% pure. Data were analyzed using FlowJo software v9.3. Antibody validation was performed by the vendors. For CyTOF, cells were stained (Supplementary Data 7) and acquired^12^ prior to analysis using DISCOV-R^6^.

### Bulk ATAC-sequencing

We performed bulk assay for transposase accessible chromatin with sequencing (ATAC-seq) on PBMC samples selected from four donors with new onset T1D that were R to alefacept (T1DAL Trial, ITN). Donors were sampled at baseline (Week 0/Visit 0) and 104 wk (Visit 30) post-treatment with alefacept. PBMC samples were stained with a flow cytometry panel (Supplementary Data 7) and sorted with the *FACS Aria II* (Becton Dickinson) cell sorter into three subsets of nonnaïve CD8^+^ T cells (singlet, live, CD14^−^ CD19^−^ CD56^-^ CD3^+^ CD8^+^ CD4^−^, not CD45RA^+^ CCR7^+^): (1) Double negative non-Tex (DN, KLRG1^−^ TIGIT^−^), (2) CD57^+^ Tex (KLRG1^+^TIGIT^+^, CD57^+^), and (3) CD57^−^ Tex (KLRG1^+^TIGIT^+^, CD57^−^) (identified as the PD-1^+^ Tex population in prior studies^12^) (Supplementary Fig. 1). Cell viability and purity were >90%. Samples were sorted into RPMI with 2% human serum and processed for bulk ATAC-seq within 2 h of sorting.

Bulk ATAC-seq was performed using the method described by ref. ^15^. Briefly, 50,000 cells were lysed to produce a crude nuclei preparation, followed by transposition and PCR amplification of the transposed DNA fragments to add Illumina-compatible indexed adapters. After 1.8x *Ampure XP* (Beckman Coulter) size selection, the concentration of each library was assessed using a 100–1000 bp gate on a Tapestation 4200 (Agilent). Pooled libraries were run with paired-end 59 base sequencing on *a NextSeq 2000* with a P3 sequencing kit (Illumina) with a target of 50 M reads.

### Bulk ATAC data processing and analysis

Following sequencing, base calls were processed to FASTQs on *BaseSpace* (Illumina), and a base call quality-trimming step was applied to remove low-confidence base calls from the ends of reads. The FASTQs were aligned to the University of California Santa Cruz (UCSC) Human genome assembly version 38.91, using *STAR* (v.2.4.2a)^53^. Duplicate filtering, QC, and metrics analysis was performed using the *Picard* (v 1.134) family of tools (https://broadinstitute.github.io/picard/). For peak calls, *MACS* (v 2.1.0) was run in paired-end mode with a *q* value cutoff of 0.05 on filtered reads^54^.

Following alignment and quality control, samples had comparable sequencing depth, and all samples exceeded 30 million reads. Fragment size distributions had clear nucleosome length peaks, and peaks were strongly enriched around the transcription start site. 23,634 total consensus open chromatin regions (OCRs) were identified that were common across samples. Differential chromatin accessibility of consensus peaks was then analyzed with *DiffBind* (v 3.4.11) in R (v 4.1.2)^55^. Peaks were counted and recentered using dba.count with duplicates removed and peak summits recentered including 1000 bp upstream and downstream of the original peak summit. Next, blacklisted regions were removed and contrasts for differential accessibility analysis were set with the DN nonnaïve cell population as the base reference, resulting in three contrasts with 8 samples each (CD57^+^ vs CD57^−^, CD57^+^ vs DN, and CD57^−^ vs DN. Samples were normalized using the recommended default *DiffBind* settings for low computational cost of library = DBA_LIBSIZE_FULL, normalize = DBA_NORM_LIBRARY, and background = FALSE. Differential accessibility was analyzed in *DiffBind* using *EdgeR*^56^ and its native TMM normalization, and sites with an FDR-adjusted p-value threshold of $\le$0.05 were considered significant^56,57^. Significant peaks were annotated to the nearest TSS, miRNA, or exon within the default binding region range of −5000 bp to 5000 bp using *ChIPpeakAnno* (v3.28.1)^58^.

Pathway enrichment and protein–protein interaction network enrichment for genes with increased differential accessibility in each contrast were calculated with *STRING* using default network settings for *Homo sapiens*^59^. Protein–protein interaction networks computed by *STRING* were plotted in R (v 4.1.2) using *igraph* (v 1.3.4), with edges plotted as combined scores calculated by *STRING* for protein–protein interaction^60^. Mean peak diagrams for individual genes (Fig. 2C) were generated using *wiggleplotR* (v 1.18) from bigwig files generated using *deepTools* without scaling (v 3.3.2)^61,62^. Drawn illustrations presented in figures were generated using *Inkscape* (v 1.2.1). Heatmaps with full ATAC peak profiles were generated using *DiffBind*. Volcano plots and bubble plots were generated using *ggplot2* (v 2 3.3.5). Scaled Venn diagrams were generated by *EulerR* (v 6.1.1)^63^.

To assess the similarity of CD57^+^ Tex cells with terminally differentiated cells (TEMRA), we first annotated significantly accessible peaks nearby genes (within 5000 bp) in CD8^+^ TEMRA cells^35^ using methods described above, and then assessed the overlap of peaks increased in accessibility in each TEMRA comparison with significantly differentially accessible peaks increased in CD57^+^ vs CD57^−^ comparison. Any amount of overlap between genomic windows across datasets determined by the *IRanges* package was considered a true overlap. We then used this overlap to calculate the Jaccard similarity coefficient (J(X,Y) = |X ∩ Y|/|X ∪ Y|), a measure of the similarity of two sets, and calculate a hypergeometric *p* value to assess enrichment.

To assess whether the percentage of TEMRA cells in the CD57^+^ Tex population affected the differential accessibility results in our ATAC-seq analysis, we took the list of differentially accessible genes increased in the CD57^+^ Tex vs the CD57^-^ (PD-1^+^ Tex) population at 104 wk and aggregated the log normalized peak score for all genes for each sample into a “CD57^+^ Tex ATAC Score”. We plotted the CD57^+^ Tex ATAC score with the percentage of TEMRA cells in each population at 104 wk that was sorted for ATAC-seq (DN, DP CD57+, DP CD57−) and measured by flow cytometry.

### Single-cell RNA sequencing

PBMCs were stained with a flow cytometry panel and sorted into non-naive CD8^+^ T cells (singlet, live, CD14^−^ CD19^−^ CD56^−^ CD3^+^ CD8^+^ CD4^−^, not CD45RA^+^ CCR7^+^) from 12 donors (six alefacept R, six NR, as defined by preservation of C-peptide levels^10,64^ with new onset T1D at 104 wk (Visit 30) post-treatment with alefacept (T1DAL Trial, ITN) using the *FACS Aria Fusion* (Becton Dickinson) cell sorter. Cell viability and purity were >90%. Samples were sorted into RPMI with 2% human serum prior to scRNA-seq. A single-cell suspension was prepared from sorted cells and loaded onto the *10x Chromium Controller* (10X Genomics) according to the manufacturer’s protocol, with a target capture of 5000 cells per channel. Sequencing libraries were generated using the NextGEM Single Cell 5’ v 1.1 kit (10x Genomics). Three pools of gene expression and TCR libraries, each from two R and two NR, were generated, with a ratio of 4:1 GEX:TCR. Each pool was run on a *NextSeq P2* flowcell on a *NextSeq 2000* sequencer (Illumina), with a target depth of 20,000 reads/cell for GEX and 5000 reads/cell for TCR libraries.

### Single-cell RNA sequencing data processing and analysis

Sequence data were processed from raw reads to gene-barcode count matrices and assembled into TCR sequences using 10x Genomics Cell Ranger v 4.0.0^65^. Reads were demultiplexed by sample and library type using index sequence pools. Gene expression reads were aligned to the human genome (GRCh38.91), and reads were collapsed to molecule counts per gene using unique molecular identifiers (UMIs). TCR sequence reads were assembled into consensus contigs, assessed for in-frame and productive sequences, and aligned to the 10x V(D)J reference v. 4.0.0 to determine gene usage. Barcodes were called as cells or backgrounds using default settings in *CellRanger* (10X), which utilizes both the distribution of UMI counts by barcode and expression comparisons of low-UMI-count barcodes to assumed background. Data from all samples were aggregated without normalization prior to downstream analysis.

10X raw data were assessed for the number of counts and features identified in each sample and the percentage of mitochondrial reads. Cells were filtered using *Seurat* (v 4) to retain only cells with greater than 200 features but less than 2500 features, and less than 25% mitochondrial reads^23^. About 22,771 genes were sequenced from 26,978 total cells. Data were then loaded into *Monocle* (v3), where cells were batch-corrected for donor ID due to the presence of several donor-specific clusters^66^. Next, Mucosal-associated Invariant T cells (MAIT) were identified by plotting the expression of CD161 (KLRB1). Cell clusters with high CD161 expression were removed from downstream analysis. Unsupervised clustering was performed using the default Leiden clustering method with a resolution of 1e-4 and dimensionality reduction (reduce_dimension) was performed using UMAP. Trajectory analysis was performed using learn_graph() with ncenter = 140 and minimal_branch_len = 6.

Following the assessment of cell state marker expression (*CCR7, PDCD1, TIGIT, KLRG1, MKI67, IL7R,* and *GZMB*) along the trajectory, cell clusters were renumbered based on order along the trajectory by putative differentiation state. To classify the phenotypes of individual clusters, the Z-score adjusted mean expression of key markers across cells in a cluster was compared between all clusters. Results were visualized in a heatmap using *ComplexHeatmap*^67^. Genes that were most specifically expressed in each cluster (top markers) as measured by the Jensen-Shannon distance were identified in *Monocle* (v3) using the top_markers function with default settings. Top markers were filtered to retain only those with a cluster specificity ≥0.25, and only the top 20 most specific markers per cluster (or less, if fewer genes met specificity criteria). Top markers retained after this procedure were expressed in at least 36% of cells in a cluster (Supplementary Data 2). Top marker normalized expression and the percentage of expression cells was plotted using plot_genes_by_group the function in Monocle (v3) to view patterns (Supplementary Fig. 9).

### *Seurat* CD8^+^ reference mapping

To better classify the phenotype of sampled CD8^+^ clusters, the *Seurat* reference mapping procedure was performed to first find anchors (normalization method = “SCT”, reference reduction = “spca”) between our dataset and the CD8^+^ T cell subset from the Seurat reference PBMC dataset^23^. Next, *MapQuery* was used to map our cells onto the reference CD8^+^ dataset UMAP space to assign inferred cell types (reference reduction = “spca”, reduction model = “wnn.umap”). Predicted cell type mapping at the L2 level was used for cell type assignments.

### scRNA-seq differential expression analysis

Differential gene expression was compared between PD-1^+^ cluster 7 and all CD57^+^ clusters 5, 6, and 8 using linear regression analysis in *Monocle* (v3) (~exhausted-like population)^66^. We also assessed differential gene expression between R and NR within the PD-1^+^ cluster 7 or CD57^+^ clusters (5, 6, and 8) using *Monocle* (v3) (~Response). Finally, differential expression was assessed to compare the combined CD57^+^ Tex clusters (5, 6, and 8) with all other clusters (~group). In all analyses, genes with an FDR-adjusted *p* value ≤ 0.05 were considered significant. Gene set enrichment between CD57^+^ Tex differentially expressed genes and previously published gene sets was assessed using hypergeometric tests (*p* values ≤ 0.05 considered significant).

### Transcription factor motif analysis

Regions of differentially accessible chromatin (identified by *DiffBind*) that either increased in accessibility in the CD57^+^ Tex population or the PD-1^+^ Tex population when compared to each other were assessed for transcription factor motif enrichment using *HOMER* (v 4.11)^36^. De novo motif enrichment results were filtered to exclude all significant hits with a *p* value > 1e-12 which were likely false positive results. Best matches identified by *HOMER* between de novo motifs and known motifs typically included several high-quality matches within the same TF motif family due to similar TF binding sites. Therefore, all potential matches of known motifs to significantly enriched de novo motifs were searched for in the list of DEGs between CD57^+^ Tex and PD-1^+^ Tex populations identified by scRNA-seq. The overlapping list of enriched TFs in accessible peaks and DEGs were plotted as a volcano plot showing their log2 fold change in gene expression and TF motif enrichment values calculated by HOMER. To assess whether these TFs were located nearby key DEGs of interest in each population and could be potential regulators of their expression, we first used the tool *GREAT* (v 4.0.4)^37^ to extend our regions of accessible chromatin and relate these regions to nearby DEGs of interest. The overlap of TF motifs of interest and DEGs near these extended accessible open chromatin regions was determined by scanning these genomic regions using the position weight matrix (PWM) for either the *TBX21* motif (for scanning CD57^+^ Tex DEGs) or the *TCF7* motif (for scanning PD-1^+^ Tex DEGs) nucleotide sequences initially identified by HOMER. PWMs for each motif and motif scanning were performed using the *universalmotif* package in R (v 1.12)^68^. TF motif hits with a *p* value ≤ 0.001 were considered significant. Protein–protein interaction networks of DEGs in each population with TF motifs for either *TBX21* (CD57^+^ Tex) or *TCF7* (PD-1^+^ Tex) were computed by *STRING* and plotted in R (v 4.1.2) using igraph (v 1.3.4), with edges plotted as combined scores calculated by *STRING* for protein–protein interaction^60^.

### TCR expansion and differentiation trajectory analysis

About 18,200 cells had a sequenced TCR chain. These cells were filtered for those with only a single alpha and single beta chain pair to remove any potential doublets and simplify downstream analysis, resulting in the retention of 5920 total cells with a full *TRA*-*TRB* pair. These cells were then mapped back onto those cells retained following *Seurat* and *Monocle* filtering, resulting in 4420 total cells with paired RNA-seq and TCR sequencing. Cell clonal expansion and sharing between clusters was determined by sharing of *TRA* chain V gene, J gene, and complementarity determining region 3 (CDR3) nucleotide (nt) sequence. Cell expansion was determined as >1 cell sharing the same *TRA* chain as defined above. This analysis was repeated using *TRB* chain sharing, and the few differences observed did not affect our results or data interpretation. For simplicity, *TRA* results are presented throughout the main text and *TRB* results are presented in the Supplementary Figs.

*TRA* sharing was plotted between clusters as a connecting line between each cell sharing their *TRA* chain sequence (“airline plot”). The sum of all these connections between clusters was plotted as a network visualization using *igraph* (v 1.3.4) with a customized plotting matrix to replicate cluster positions in the scRNA-seq UMAP dimensionality reduction. Connections between individual pairs of clusters was also plotted as a heatmap using *ComplexHeatmap* (v.2.1.0)^67^. The total number of connections between each potential precursor cluster (1, 2, 3, or 4) with all the exhausted-like cell clusters (5, 6, 7, 8) for each individual donor were statistically compared using a Kruskal–Wallis one-way analysis of variance. The overlap of *TRA* repertoires (the unique *TRA* chains in each cluster), between each cluster were determined by calculating the Jaccard similarity coefficient (J(X,Y) = |X ∩ Y|/|X∪Y|) for each combination of cluster repertoires^69^. This coefficient describes the similarity between two lists and is presented on a scale of 0-1, with 0 indicating no sharing between lists, and 1 indicating complete sharing. Calculated Jaccard similarities between clusters were plotted as a heatmap to identify groups of clusters with similar Jaccard index values using ComplexHeatmap (v 2.1.0)^67^.

Unique combinations of *TRA* chains between clusters were identified using the *Vennerable* package (v3.1). Distinct combinations of *TRA* sharing were plotted as an upset plot using *ComplexHeatmap* (v.2.1.0). Distinct combinations of cluster sharing were then categorized by cluster phenotype into four distinct differentiation trajectories. Cells with *TRA*s in each distinct differentiation trajectory were plotted using a UMAP dimensionality reduction. Shannon-Wiener diversity of *TRA*s with each differentiation trajectory was calculated per donor using the vegan package (v 2.5-5)^70^. All cartoons in all manuscript figures presented in the paper were generated using *Inkscape* (v 1.2.1).

*TRA* and *TRB* chain antigen specificities were determined by matching the V gene, J gene, CDR3 amino acid sequence, and chain to hits in the VDJdb (downloaded March 20, 2022) curated database of TCR sequences with known antigen specificities^30^. Cells with *TRA* sequences matching to either CMV or EBV antigen species were analyzed for their differentiation trajectories across the UMAP dimensionality reduction plot and compared to percentages of CMV and EBV-specific cells of either PD-1^+^ Tex and CD57^+^ Tex population identified by flow cytometry for the same patients.

### Statistics and reproducibility

Statistical tests were performed using the R programming language and software environment. We utilized t-tests for group comparisons of continuous, normally distributed variables; Wilcoxon signed rank tests for non-normally distributed variables; the hypergeometric test to determine the significance of set overlaps; Fisher’s exact test for categorical variables; and linear modeling for line slopes. Unless otherwise noted, we performed non-paired, two-sided tests and assumed equivalent variation in the groups compared. Where appropriate, multiple testing corrections were made. Linear regressions show standard errors at the 0.95 confidence level. Goodness of fit for linear regression lines was indicated by Pearson corrrelation coefficients. Boxplots show five summary statistics (the median, two hinges at the 25th and 75th percentiles, and two whiskers showing ±1.5 * the interquartile range). Error bars represent standard errors. The term “significant” is reserved for *p* values (single tests) or false-discovery rate (FDR)-adjusted *p* values (*p*Adj) (multiple tests) of <0.05. Specific tests used to derive each listed *p* value are given in the text or in the Figure legends. The reproducibility of experiments was judged by testing samples from different individuals, and together with the sample sizes, is indicated by points showing data distribution.

### Supplementary information

Supplementary Information

Description of Additional Supplementary Files

Supplementary Data 1

Supplementary Data 2

Supplementary Data 3

Supplementary Data 4

Supplementary Data 5

Supplementary Data 6

Supplementary Data 7

Supplementary Data 8

## Data Availability

Supplementary Data tables, including Source Data, are available publicly on figshare (https://figshare.com/projects/Interconnected_lineage_trajectories_link_conventional_and_NK-like_exhausted-like_CD8_T_cells_beneficial_in_T1D/203754). The source data behind the graphs in the paper can be found in Supplementary Data 8 in the figshare submission linked above. scRNA-seq, TCR data, and ATAC-seq data analyzed for this publication are publicly available in GEO SuperSeries GSE237614. Cytometry data are available to freely registered users in ITN TrialShare (https://www.itntrialshare.org/project/Studies/ITN045AIPUBLIC/Study%20Data/begin.view?pageId=study.DATA_ANALYSIS).

## Code availability

Code and data used to perform analyses and generate figures is shared publicly without restrictions on GitHub (https://github.com/BenaroyaResearch/Witkop_T1DAL_2023).

## References

[1] Wherry EJ, Kurachi M (2015). Molecular and cellular insights into T cell exhaustion. Nat. Rev. Immunol..

[2] Collier JL, Weiss SA, Pauken KE, Sen DR, Sharpe AH (2021). Not-so-opposite ends of the spectrum: CD8(+) T cell dysfunction across chronic infection, cancer and autoimmunity. Nat. Immunol..

[3] Beltra J-C (2020). Developmental relationships of four exhausted CD8+ T cell subsets reveals underlying transcriptional and epigenetic landscape control mechanisms. Immunity.

[4] Blank CU (2019). Defining ‘T cell exhaustion. Nat. Rev. Immunol..

[5] McKinney EF, Lee JC, Jayne DR, Lyons PA, Smith KG (2015). T-cell exhaustion, co-stimulation and clinical outcome in autoimmunity and infection. Nature.

[6] Wiedeman AE (2020). Autoreactive CD8+ T cell exhaustion distinguishes subjects with slow type 1 diabetes progression. J. Clin. Invest..

[7] Linsley PS, Long SA (2019). Enforcing the checkpoints: harnessing T-cell exhaustion for therapy of T1D. Curr. Opin. Endocrinol. Diabetes Obes..

[8] Belk JA, Daniel B, Satpathy AT (2022). Epigenetic regulation of T cell exhaustion. Nat. Immunol..

[9] Daniel B (2022). Divergent clonal differentiation trajectories of T cell exhaustion. Nat. Immunol..

[10] Long, S. A. et al. Partial exhaustion of CD8 T cells and clinical response to teplizumab in new-onset type 1 diabetes. *Sci. Immunol.* **1**, eaai7793 (2016). 10.1126/sciimmunol.aai7793 PMC5486405 28664195

[11] Herold KC (2019). An anti-CD3 antibody, teplizumab, in relatives at risk for type 1 diabetes. N. Engl. J. Med..

[12] Diggins, K. E. et al. Exhausted-like CD8+ T cell phenotypes linked to C-peptide preservation in alefacept-treated T1D subjects. *JCI Insight* **6**, e142680 (2021). 10.1172/jci.insight.142680 PMC7934874 33351781

[13] Li J (2022). KIR(+)CD8(+) T cells suppress pathogenic T cells and are active in autoimmune diseases and COVID-19. Science.

[14] Kwong CJ (2021). Harnessing CD8(+) T-cell exhaustion to treat type 1 diabetes. Immunol. Cell Biol..

[15] Buenrostro JD, Wu B, Chang HY, Greenleaf WJ (2015). ATAC-seq: a method for assaying chromatin accessibility genome-wide. Curr. Protoc. Mol. Biol..

[16] Rigby MR (2015). Alefacept provides sustained clinical and immunological effects in new-onset type 1 diabetes patients. J. Clin. Invest..

[17] Yates KB (2021). Epigenetic scars of CD8(+) T cell exhaustion persist after cure of chronic infection in humans. Nat. Immunol..

[18] Giles JR (2022). Shared and distinct biological circuits in effector, memory and exhausted CD8(+) T cells revealed by temporal single-cell transcriptomics and epigenetics. Nat. Immunol..

[19] Grebinoski S (2022). Autoreactive CD8(+) T cells are restrained by an exhaustion-like program that is maintained by LAG3. Nat. Immunol..

[20] Sullivan BM, Juedes A, Szabo SJ, von Herrath M, Glimcher LH (2003). Antigen-driven effector CD8 T cell function regulated by T-bet. Proc. Natl Acad. Sci. USA.

[21] Nielsen CM, White MJ, Goodier MR, Riley EM (2013). Functional significance of CD57 expression on human NK cells and relevance to disease. Front. Immunol..

[22] Dufort, M. J., Greenbaum, C. J., Speake, C. & Linsley, P. S. Cell type-specific immune phenotypes predict loss of insulin secretion in new-onset type 1 diabetes. *JCI Insight* **4**, e125556 (2019). 10.1172/jci.insight.125556 PMC6478408 30830868

[23] Hao Y (2021). Integrated analysis of multimodal single-cell data. Cell.

[24] Zheng L (2021). Pan-cancer single-cell landscape of tumor-infiltrating T cells. Science.

[25] Wherry EJ (2007). Molecular signature of CD8+ T cell exhaustion during chronic viral infection. Immunity.

[26] Pearce EL (2003). Control of effector CD8+ T cell function by the transcription factor eomesodermin. Science.

[27] Intlekofer AM (2005). Effector and memory CD8+ T cell fate coupled by T-bet and eomesodermin. Nat. Immunol..

[28] Sottile R (2021). Human cytomegalovirus expands a CD8+ T cell population with loss of BCL11B expression and gain of NK cell identity. Sci. Immunol..

[29] Rodriguez, I. J. et al. Immunosenescence study of T cells: a systematic review. *Front. Immunol.* **11**, 604591 (2021). 10.3389/fimmu.2020.604591 PMC7843425 33519813

[30] Saul D (2022). A new gene set identifies senescent cells and predicts senescence-associated pathways across tissues. Nat. Commun..

[31] Purcell M, Kruger A, Tainsky MA (2014). Gene expression profiling of replicative and induced senescence. Cell Cycle.

[32] Fridman AL, Tainsky MA (2008). Critical pathways in cellular senescence and immortalization revealed by gene expression profiling. Oncogene.

[33] Gillespie M (2022). The reactome pathway knowledgebase 2022. Nucleic Acids Res..

[34] Zheng H-Y (2020). Elevated exhaustion levels and reduced functional diversity of T cells in peripheral blood may predict severe progression in COVID-19 patients. Cell. Mol. Immunol..

[35] Giles JR (2022). Human epigenetic and transcriptional T cell differentiation atlas for identifying functional T cell-specific enhancers. Immunity.

[36] Heinz S (2010). Simple combinations of lineage-determining transcription factors prime cis-regulatory elements required for macrophage and B cell identities. Mol. Cell.

[37] McLean CY (2010). GREAT improves functional interpretation of cis-regulatory regions. Nat. Biotechnol..

[38] Pauken KE, Wherry EJ (2015). Overcoming T cell exhaustion in infection and cancer. Trends Immunol..

[39] de Melo Silva J, Pinheiro-Silva R, Dhyani A, Pontes GS (2020). Cytomegalovirus and Epstein-Barr infections: prevalence and impact on patients with hematological diseases. Biomed. Res. Int..

[40] Shugay M (2018). VDJdb: a curated database of T-cell receptor sequences with known antigen specificity. Nucleic Acids Res..

[41] Abdel-Hakeem MS (2021). Epigenetic scarring of exhausted T cells hinders memory differentiation upon eliminating chronic antigenic stimulation. Nat. Immunol..

[42] Pauken KE (2016). Epigenetic stability of exhausted T cells limits durability of reinvigoration by PD-1 blockade. Science.

[43] Kasmani, M. Y. et al. Clonal lineage tracing reveals mechanisms skewing CD8+ T cell fate decisions in chronic infection. *J. Exp. Med.* **220**, e20220679 (2023). 10.1084/jem.20220679 PMC9623343 36315049

[44] Chen Z (2019). TCF-1-centered transcriptional network drives an effector versus exhausted CD8 T cell-fate decision. Immunity.

[45] Pritykin Y (2021). A unified atlas of CD8 T cell dysfunctional states in cancer and infection. Mol. Cell.

[46] Zander R, Cui W (2023). Exhausted CD8(+) T cells face a developmental fork in the road. Trends Immunol..

[47] Levescot A, Cerf-Bensussan N (2022). Regulatory CD8(+) T cells suppress disease. Science.

[48] Duraiswamy J (2011). Phenotype, function, and gene expression profiles of programmed death-1(hi) CD8 T cells in healthy human adults. J. Immunol..

[49] Bhatt RS (2021). KIR3DL3 is an inhibitory receptor for HHLA2 that mediates an alternative immunoinhibitory pathway to PD1. Cancer Immunol. Res..

[50] Su Q, Du J, Xiong X, Xie X, Wang L (2023). B7-H7: a potential target for cancer immunotherapy. Int. Immunopharmacol..

[51] Diggins KE, Greenplate AR, Leelatian N, Wogsland CE, Irish JM (2017). Characterizing cell subsets using marker enrichment modeling. Nat. Methods.

[52] Hirsch JS (2023). FDA approves teplizumab: a milestone in type 1 diabetes. Lancet Diabetes Endocrinol..

[53] Dobin A (2013). STAR: ultrafast universal RNA-seq aligner. Bioinformatics.

[54] Zhang Y (2008). Model-based analysis of ChIP-seq (MACS). Genome Biol..

[55] Ross-Innes CS (2012). Differential oestrogen receptor binding is associated with clinical outcome in breast cancer. Nature.

[56] Robinson MD, McCarthy DJ, Smyth GK (2010). edgeR: a Bioconductor package for differential expression analysis of digital gene expression data. Bioinformatics.

[57] Robinson MD, Oshlack A (2010). A scaling normalization method for differential expression analysis of RNA-seq data. Genome Biol..

[58] Zhu LJ (2010). ChIPpeakAnno: a Bioconductor package to annotate ChIP-seq and ChIP-chip data. BMC Bioinformatics.

[59] Szklarczyk D (2019). STRING v11: protein-protein association networks with increased coverage, supporting functional discovery in genome-wide experimental datasets. Nucleic Acids Res..

[60] Csardi G, Nepusz T (2006). The igraph software package for complex network research. InterJournal Complex Syst..

[61] Alasoo, K. wiggleplotr: make read coverage plots from BigWig files. *Bioconductor*. **10**, B9 (2017).

[62] Ramirez F (2016). deepTools2: a next generation web server for deep-sequencing data analysis. Nucleic Acids Res..

[63] Larsson, J. eulerr: area-proportional Euler and Venn diagrams with ellipses. 783 (2022).

[64] Sims, E. K. et al. Teplizumab improves and stabilizes beta cell function in antibody-positive high-risk individuals. *Sci. Transl. Med.* **13**, eabc8980 (2021). 10.1126/scitranslmed.abc8980 PMC8610022 33658358

[65] Zheng GX (2017). Massively parallel digital transcriptional profiling of single cells. Nat. Commun..

[66] Trapnell C (2014). The dynamics and regulators of cell fate decisions are revealed by pseudotemporal ordering of single cells. Nat. Biotechnol..

[67] Gu Z, Eils R, Schlesner M (2016). Complex heatmaps reveal patterns and correlations in multidimensional genomic data. Bioinformatics.

[68] Tremblay, B. J. universalmotif: import, modify, and export motifs with R. https://bioconductor.org/packages/universalmotif/ (2022).

[69] Vorontsov IE, Kulakovskiy IV, Makeev VJ (2013). Jaccard index based similarity measure to compare transcription factor binding site models. Algorithms Mol. Biol..

[70] Oksanen, J. et al. Vegan: community ecology package, R package version 2.6-4. https://CRAN.R-project.org/package=vegan (2022).

