# Supplementary material for: Interconnected lineage trajectories link conventional and natural killer (NK)-like exhausted CD8+ T cells beneficial in type 1 diabetes
Source: Commun Biol. 2024 Jun 27;7:773. doi: 10.1038/s42003-024-06456-3 (PMC11211332; doi:10.1038/s42003-024-06456-3)
Supplement: Supplementary file 1 — Supplementary Information [file 42003_2024_6456_MOESM1_ESM.pdf]

Supplementary Information

Interconnected lineage trajectories link conventional and Natural Killer (NK)-like exhausted $CD8^+$ T cells beneficial in type 1 diabetes

Erin M. Witkop[1], Kirsten Diggins[1], Alice Wiedeman[2], Elisavet Serti[3], Gerald Nepom[2,3,] Vivian H. Gersuk[4], Bryce Fuchs[2], S. Alice Long[2]**, Peter S. Linsley[1]**

**co-senior author
1 Benaroya Research Institute, Systems Immunology, 1201 Ninth Avenue, Seattle, WA 98101USA
2 Benaroya Research Institute, Translational Immunology, 1201 Ninth Avenue, Seattle, WA USA98101
3 Immune Tolerance Network (ITN), Bethesda, MarylandMD, USA
4 Benaroya Research Institute, Genomics Core, 1201 Ninth Avenue, Seattle, WA USA98101

** These authors jointly supervised this work

Corresponding author: plinsley@benaroyaresearch.org

Supplementary Figures

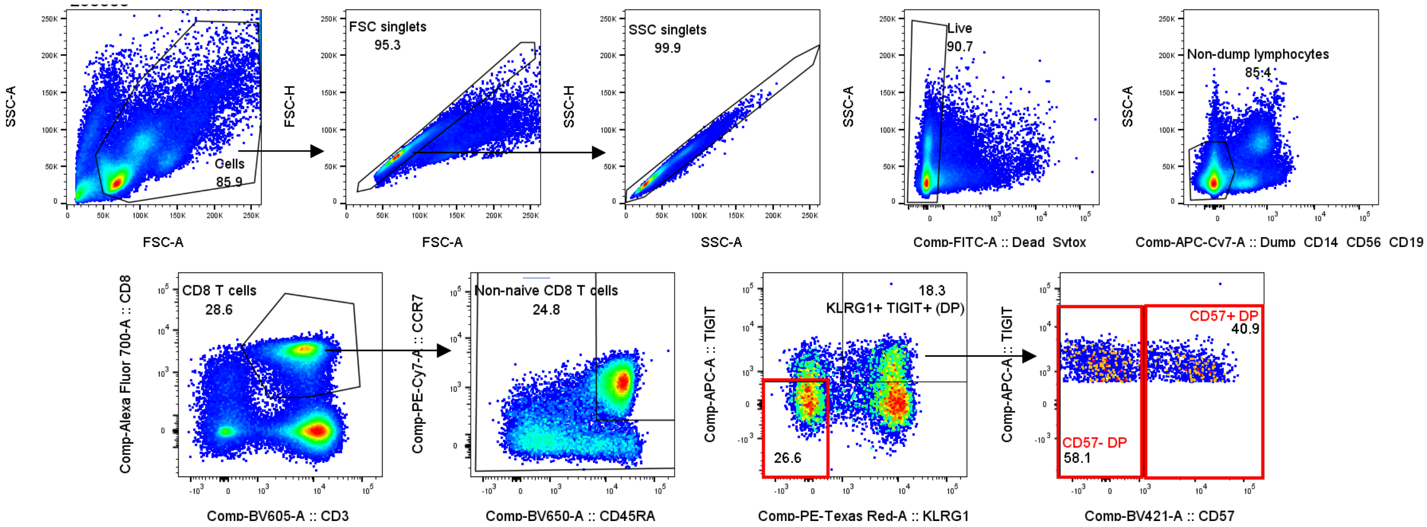

Supplementary Figure 1. Flow cytometry gating scheme used to sort PBMC into CD8+ TIGIT+KLRG1+ CD57+ Tex and CD57- (PD-1+) Tex populations for bulk ATAC-sequencing. n=4 R, Responders, Visit 0 = baseline, Visit 30 = 104 wk post-treatment.

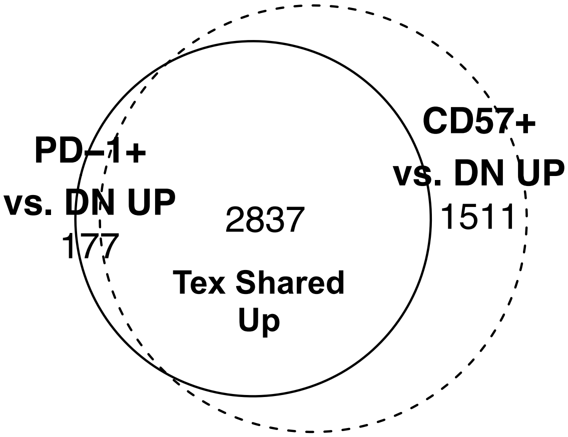

Supplementary Figure 2. Most differentially accessible sites in PD-1^{+} Tex vs DN and CD57^{+} Tex vs DN overlap. Scaled Venn diagram showing the overlap between differentially accessible chromatin sites in each population that had significantly increased chromatin accessibility versus the control TIGIT KLRG1 double negative (DN) population ("Tex Shared Up") (n=4 Responders, sampled at Visit 0, baseline, and Visit 30, 104 wk post-treatment).

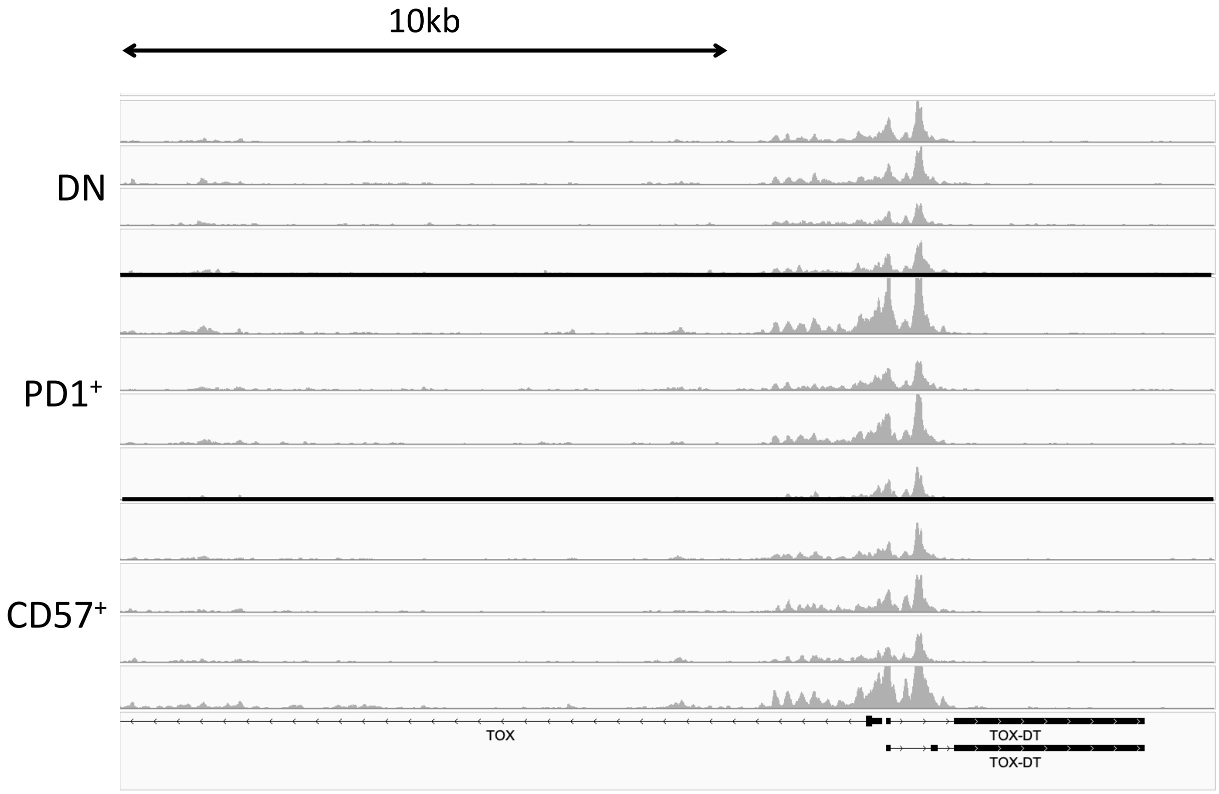

Supplementary Figure 3. Open chromatin upstream of the *TOX* gene in DN, PD1⁺ and CD57⁺ cell populations. Shown is ATAC-seq read coverage near the 5'end of the *TOX* gene (Chr 8: 58.81 – 59.12).

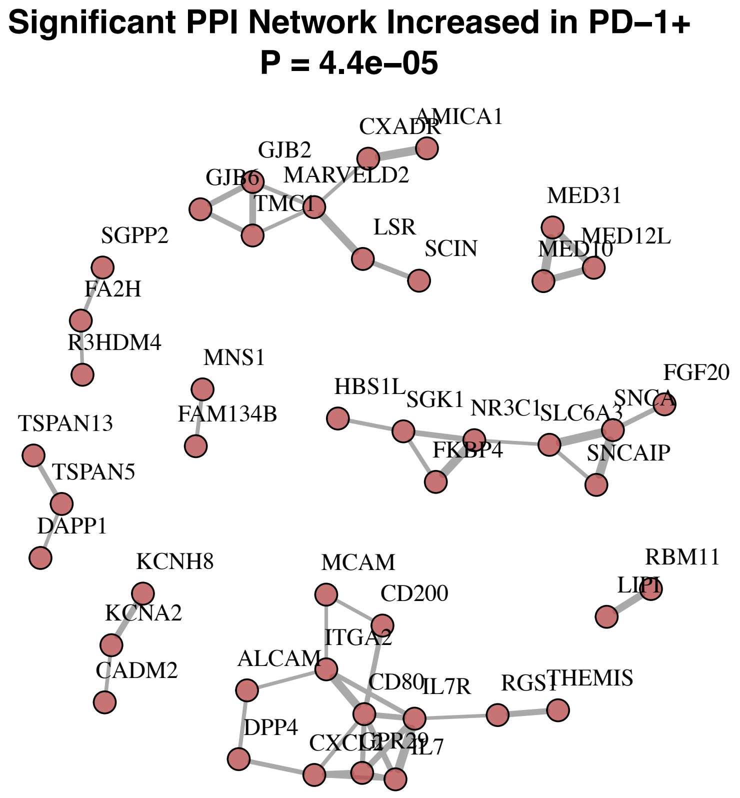

Supplementary Figure 4. Enriched protein-protein interaction network of increased differentially accessible genes in the PD-1⁺ Tex population. Enriched protein-protein interaction network among genes with increased differential accessibility in the PD-1⁺ Tex population vs. the CD57⁺ Tex population, as determined by StringDB (n=4 Responders, sampled at Visit 0, baseline, and Visit 30, 104 wk post-treatment). The significantly enriched protein-protein network was plotted using *igraph*[1] with edges weighted by the strength of protein-protein interactions. The PPI enrichment p-value was calculated using *STRING*.

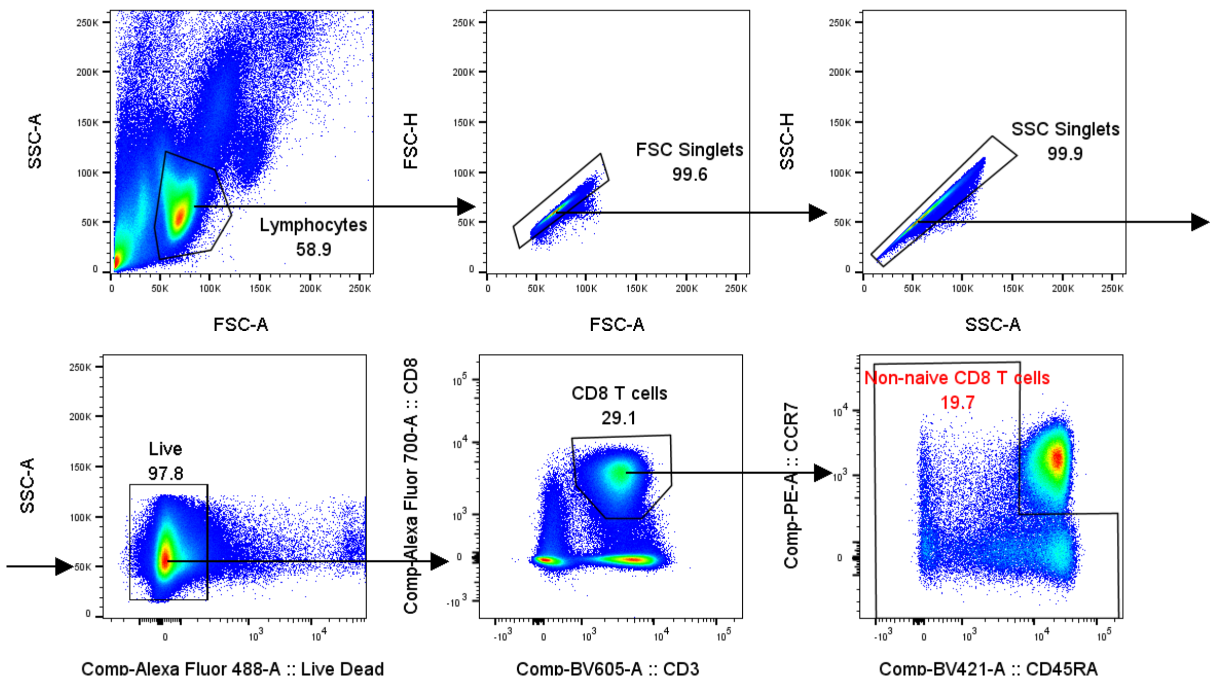

Supplementary Figure 5. Flow cytometry gating scheme used to sort non-naïve CD8⁺ T cells from PBMC for scRNA-sequencing. n = 12 donors sampled for dual scRNA-seq and TCR-seq, 6 R, 6 NR, 104 wk post-treatment.

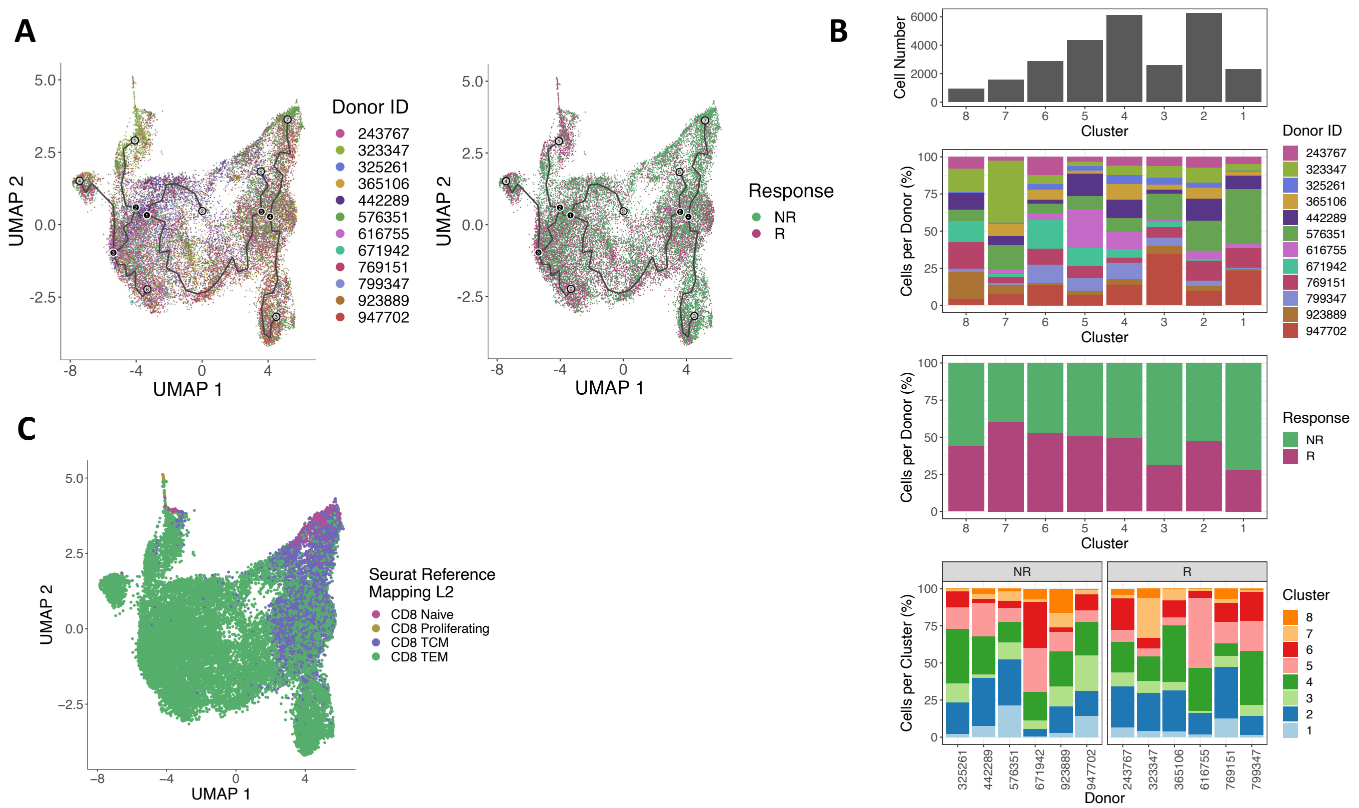

Supplementary Figure 6. scRNA-seq reveals even donor mixing across clusters and reference CD8⁺ phenotypes. a) UMAP dimensionality reduction showing all cells colored by their donor ID and overlayed with the monocle trajectory (n=12, 6 R, 6 NR sampled at 104 wk post-treatment). b) The number of total cells per cluster (top) and the proportion of cells from each donor (separated by response group) per cluster vary (bottom), but multiple donors are represented in each cluster (middle). c) scRNA-seq data was mapped to the Seurat $CD8^+$ Reference dataset at level 2 (L2), identifying cells in the trajectory as most similar to reference $CD8^+$ naïve, proliferating, T_{CM} and T_{EM} phenotypes.

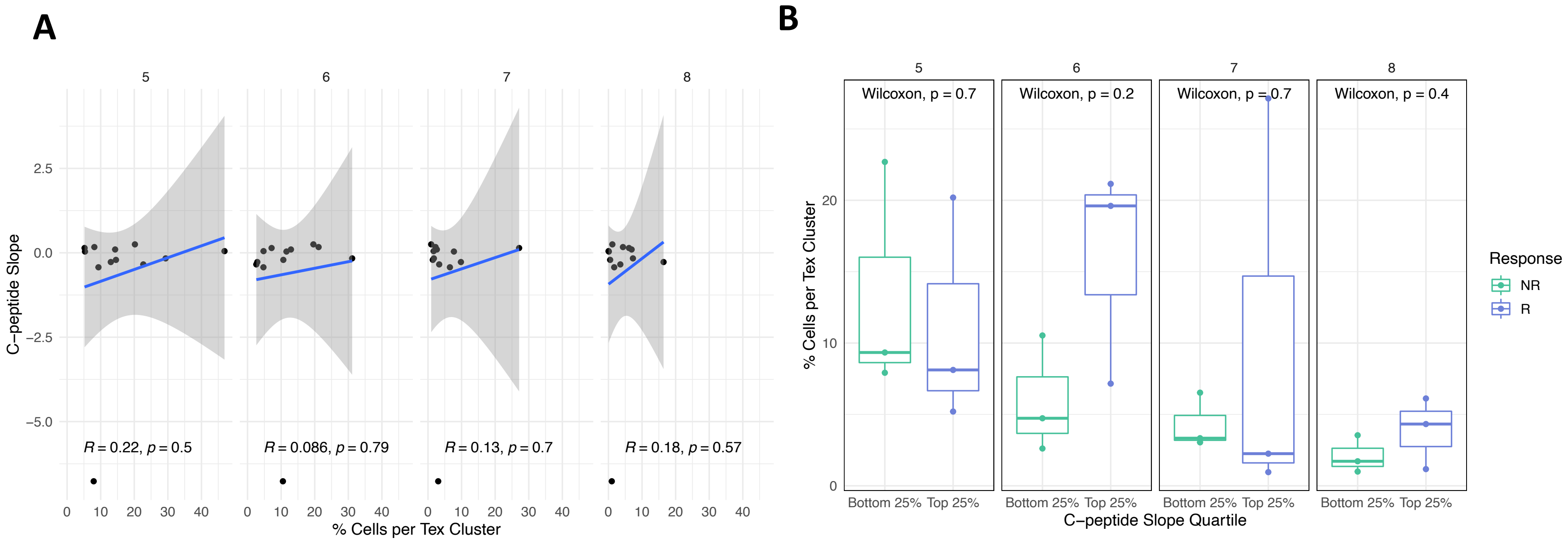

Supplementary Figure 7. Association between C-peptide slope and percent of cells in each Tex scRNA-seq cluster. The rate of loss of C-peptide (C-peptide slope)[2] was plotted versus the number of cells in T cell clusters identified as exhausted. a) The percentage of cells in each scRNA-seq cluster was plotted against the calculated C-peptide slope for each patient. The percentage of cells was not significantly correlated with C-peptide slope in any Tex cluster. P-values were calculated using linear modeling. Pearson's correlation coefficients were used. b) Patients were stratified by the top and bottom quartile of C-peptide slope and plotted with the percent of cells in each cluster. Boxes extend from the first to the third quartiles (interquartile range) with a line in the middle that represents the median. Lines extending from the boxes (whiskers) represent variability outside the interquartile range. Dots represent values from individual donors.

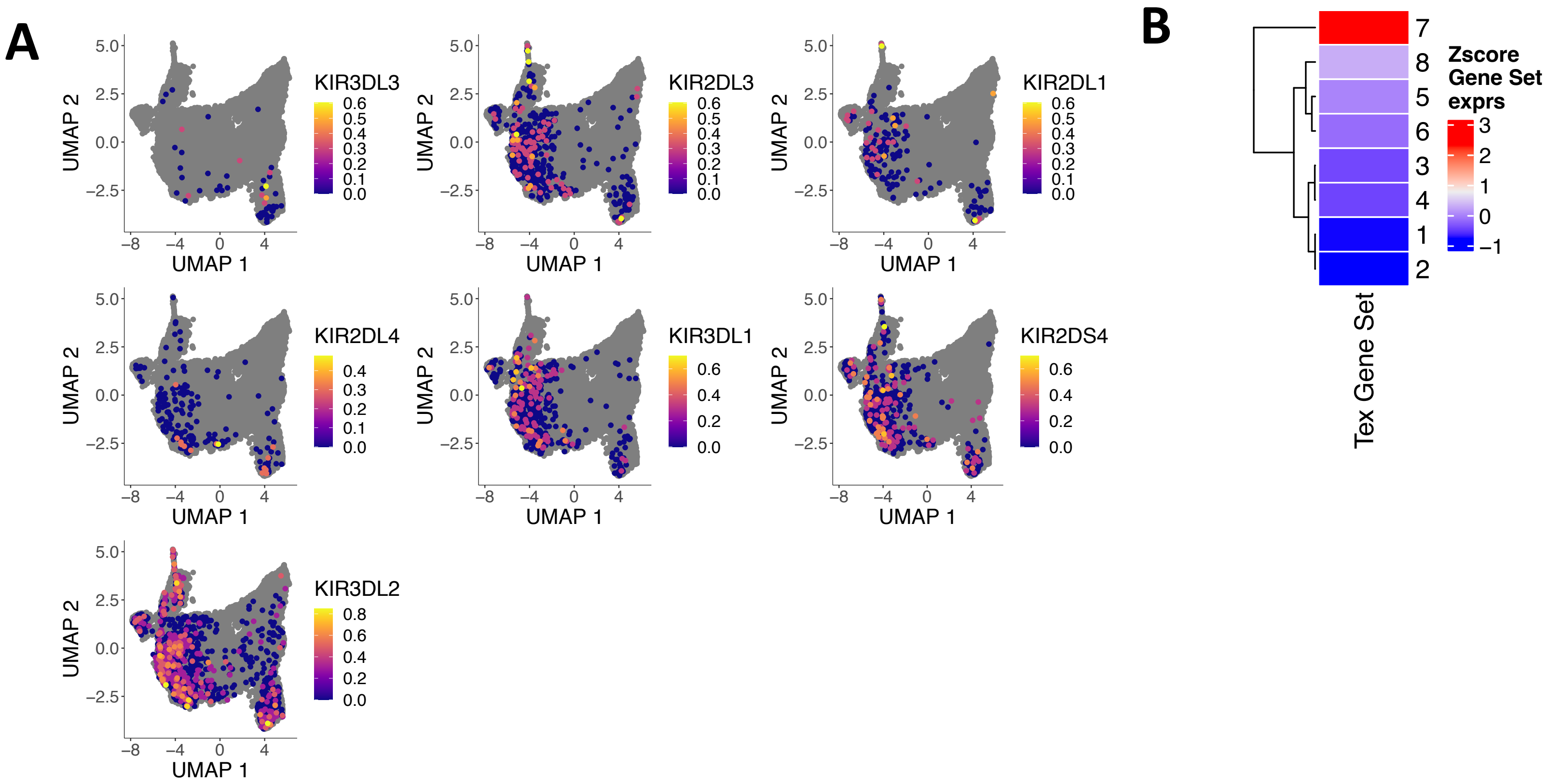

Supplementary Figure 8. More terminal CD8⁺ clusters 5, 6, 7, and 8 express exhaustion-associated genes and KIR genes. a) Log10 normalized single cell gene expression of KIR family genes displayed on a UMAP dimensionality reduction showing KIR greater expression in more terminal clusters 5, 6, 7 and 8. b) Heatmap showing Z-score scaled mean expression of all key exhaustion markers from Figure 3D.

A

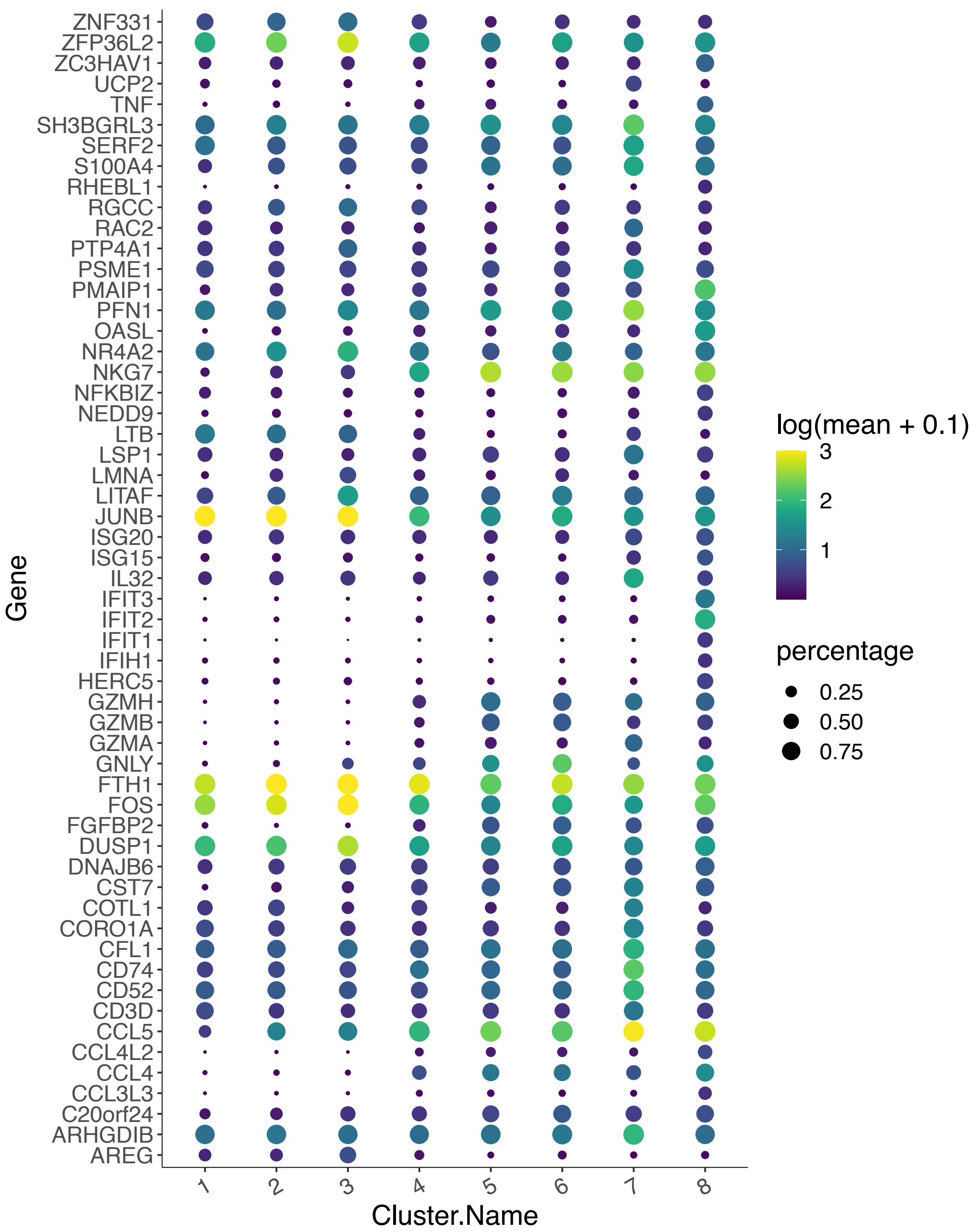

Supplementary Figure 9. Scaled expression of top 10 markers differentiating each cluster. The top 10 markers whose expression differentiated each cluster from the others were identified in *Monocle* (specificity $\geq$ 0.25*)* (n=12, 6 R, 6 NR sampled at 104 wk post-treatment).

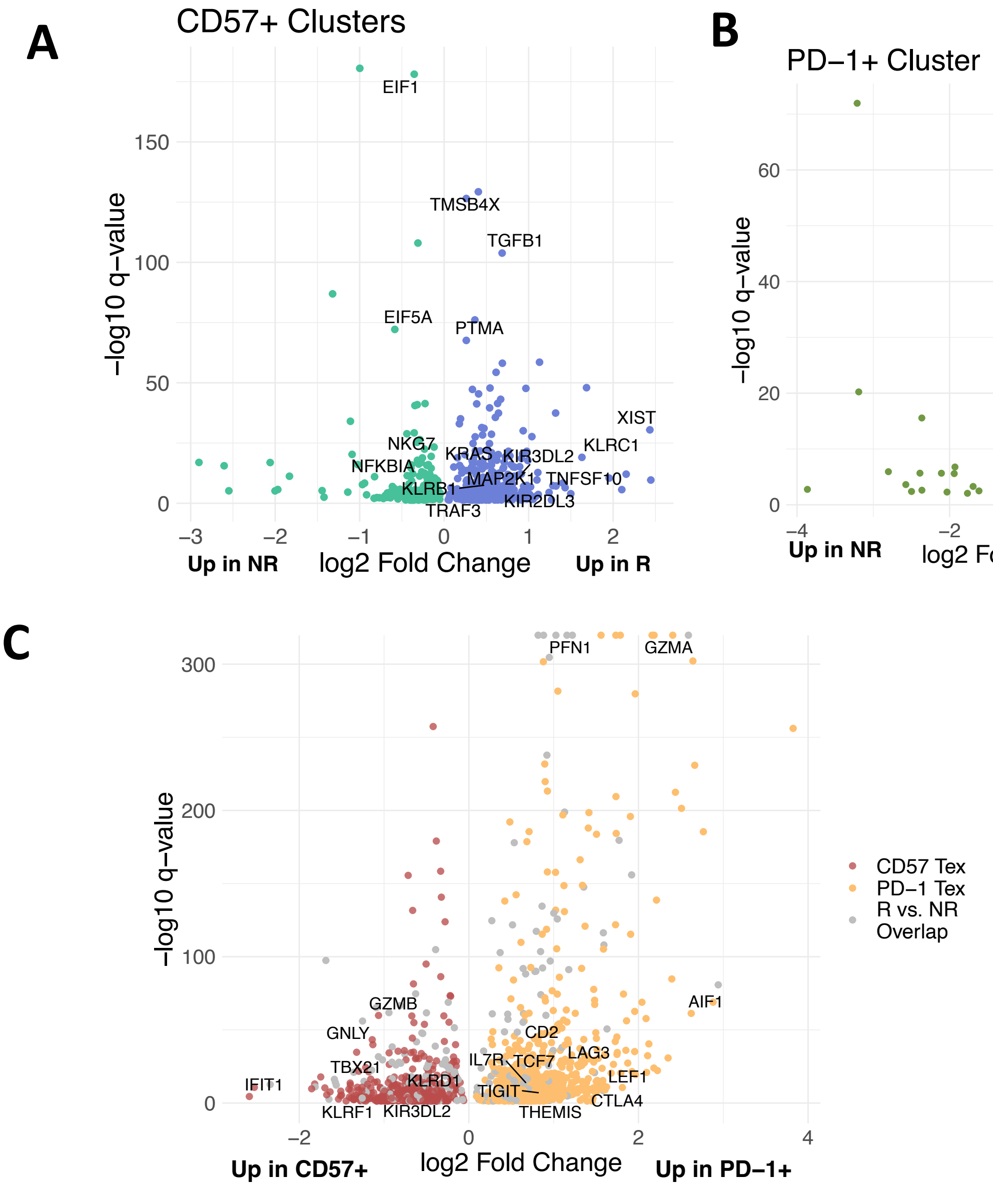

Supplementary Figure 10. Differential expression between responders and non-responders in PD-1^{+} Tex and CD57^{+}_ Tex clusters. a) log2 fold change and -log10 q-value of significantly differentially expressed genes (q-value ≤ 0.05) in R vs. NR within the combined CD57^{+} Tex clusters (5, 6, 8). b) R vs. NR within PD-1^{+} Tex cluster (7). c) differentially expressed genes (q-value ≤ 0.05) between the PD-1^{+} Tex cluster and the combined CD57^{+} Tex clusters, with genes plotted in gray if they were also significantly differentially expressed within a given cell population between R vs NR patients. This indicates that the expression of several highly significant genes was influenced by drug response.

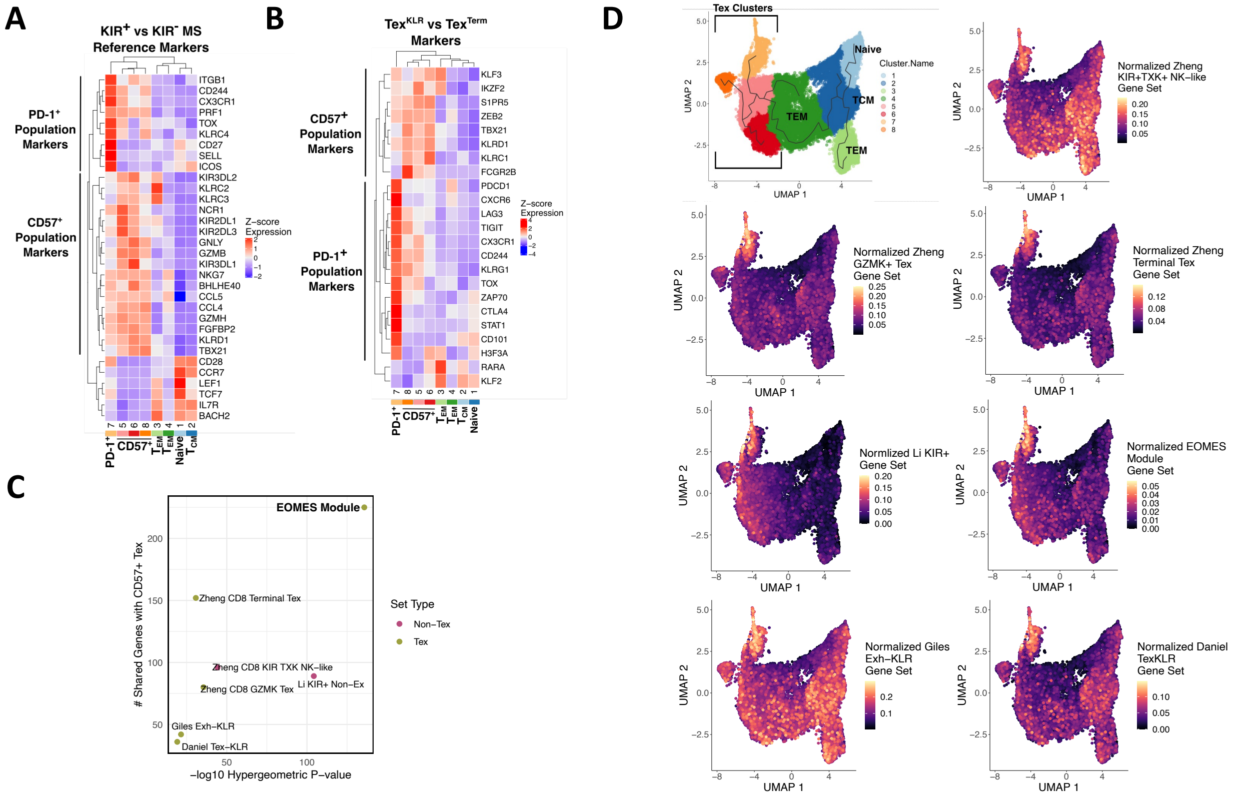

Supplementary Figure 11. NK-like gene sets differentiate PD-1^{+} and CD57^{+} Tex clusters. a) Unsupervised heatmap clustering of Z-score adjusted mean gene expression per cluster (n = 12 donors sampled for dual scRNA-seq and TCR-seq, 6 R, 6 NR, 104 wk post-treatment) using the gene list from Li et al.[3] (their Supplementary Figure 4a). This gene list differentiates KIR^{+} and KIR^{-} regulatory CD8^{+} T cells. b) Clustering using the published gene list from Daniel et al. (their Figure 2b) distinguishing the Tex^{Term} and Tex^{KLR} CD8^{+} T cell subsets[4]. Though mice possess Killer Lectin-Like Receptors but not Killer Ig-like Receptor genes, expression of NK-associated receptors characterizing the Tex^{KLR} population also characterize clustering of our CD57^{+} clusters. c) Hypergeometric p-value versus the number of genes shared between DEGs that were increased in CD57^{+} Tex clusters (**Supplementary Table 6**) and published gene sets that characterized Tex or NK-like cells[3,5–8]. d) UMAP dimensionality reduction plots showing the aggregated expression of all genes in published gene sets presented in (c).

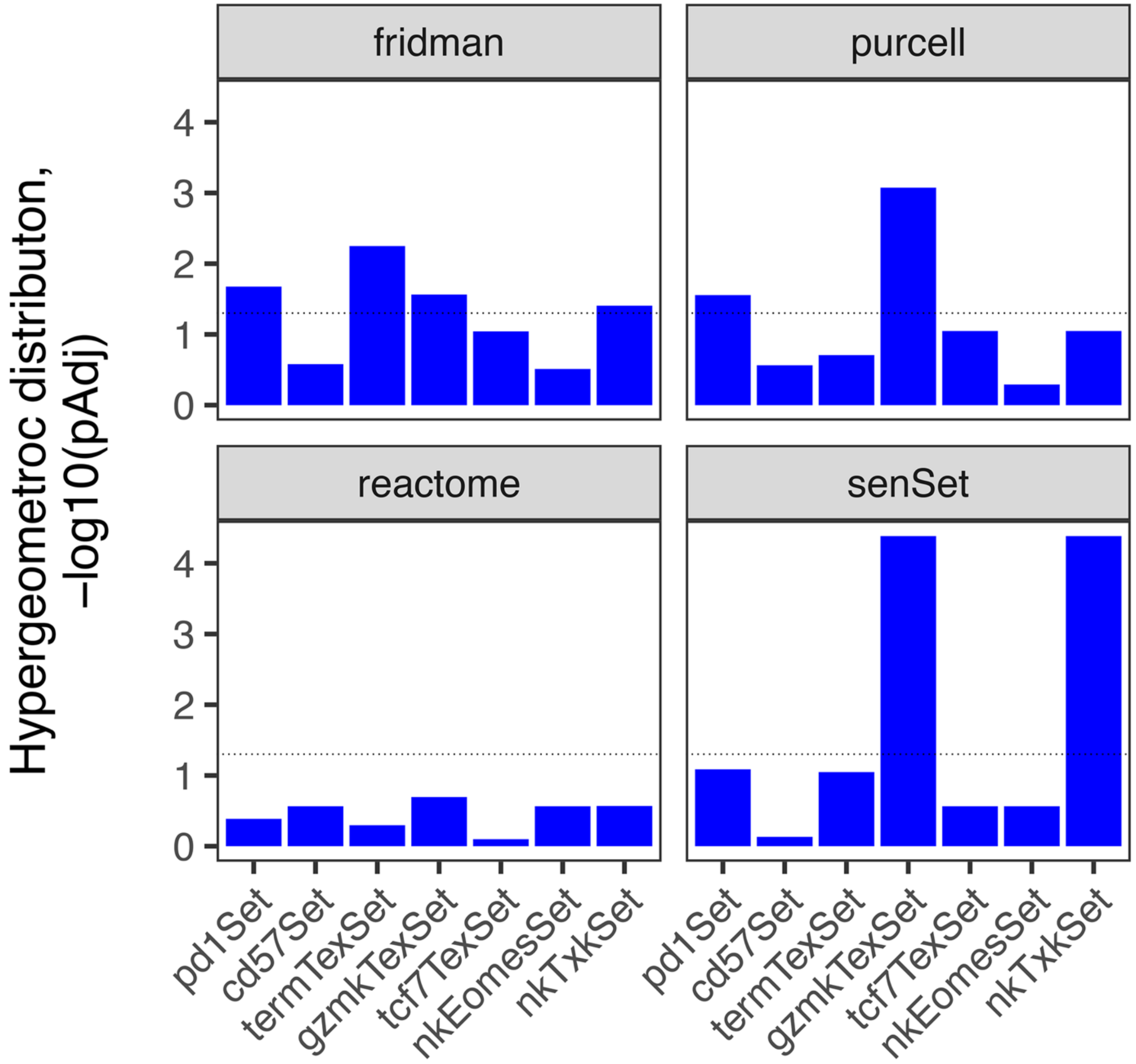

Supplementary Figure 12. Senescence gene sets do not overlap significantly with PD-1$^+$ and CD57$^+$ Tex clusters. Plotted are -log10 values of the adjusted hypergeometric p-values for enrichment of senescent gene sets: fridman[9]; purcell[10]; reactome[11] and SenSet[12] in other gene sets. Other gene sets included: pd1Set, DEGs that were increased in PD1+$^+$ cluster 7 versus CD57+ Tex clusters 5,6,and 8 (**Supplementary Table 3);** cd57Set, DEGs that were increased in CD57$^+$cluster 7 versus PD1+ Tex clusters 7 (**Supplementary Table 3**); and termTexSet, gzmkTexSet, tcf7TexSet, nkEomesSet and nkTxSet gene sets that characterized Tex or NK-like cells[5]. A"universe" of 15,900 genes was used. Dotted line, adjusted hypogeometric p-value equal to 0.05.

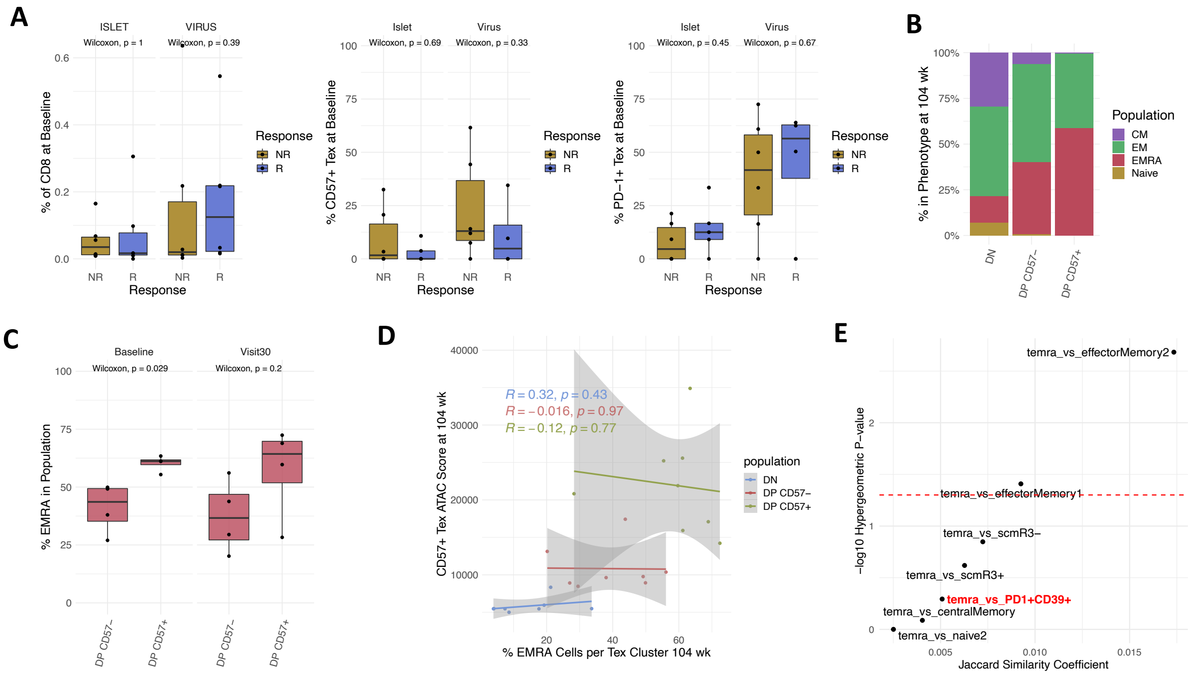

Supplementary Figure 13. Lack of significant relationship between Tex populations and antigen specificity or TEMRA differentiation. a) The percent of total CD8^{+} T cells, CD57^{+} Tex or PD-1^{+} Tex T cells with either pooled islet or chronic viral antigen[13]. Boxes extend from the first to the third quartiles (interquartile range) with a line in the middle that represents the median. Lines extending from the boxes (whiskers) represent variability outside the interquartile range. Wilcoxon rank sum p-values were used. b) The percent of Naïve, CM, EM, and TEMRA cells in each population sorted for ATAC-seq (**Figure 1**). c) Percent of TEMRA (EMRA) cells in TIGIT^{+}KLRG1^{+} (DP) CD57^{+} Tex and PD-1^{+} Tex populations at Baseline and 104 wk. The percent of TEMRA cells did not differ between did not differ between visits. Wilcoxon rank sum p-values were used. d) CD57^{+} Tex ATAC “score” (Methods) versus the percentage of TEMRA cells in each population for each donor at 104 wk. The percent of TEMRA cells in each population did not correlate with the CD57+ Tex ATAC score. P-values were from linear modeling. Pearson’s correlation coefficients were used. Gray areas represent 95% confidence intervals. e) Enrichment of TEMRA differentially accessible ATAC-seq peaks from Giles et al.[8] in the CD57^{+} Tex vs PD-1^{+} differentially accessible peaks, measured by hypergeometric p-values and Jaccard similarity coefficients (J(X,Y) = |X∩Y| / |X∪Y|). The most applicable comparison to assess similarity with CD57^{+} Tex and PD-1^{+} Tex was the “temra_vs_PD1+CD39+” gene set, which was not significantly enriched.

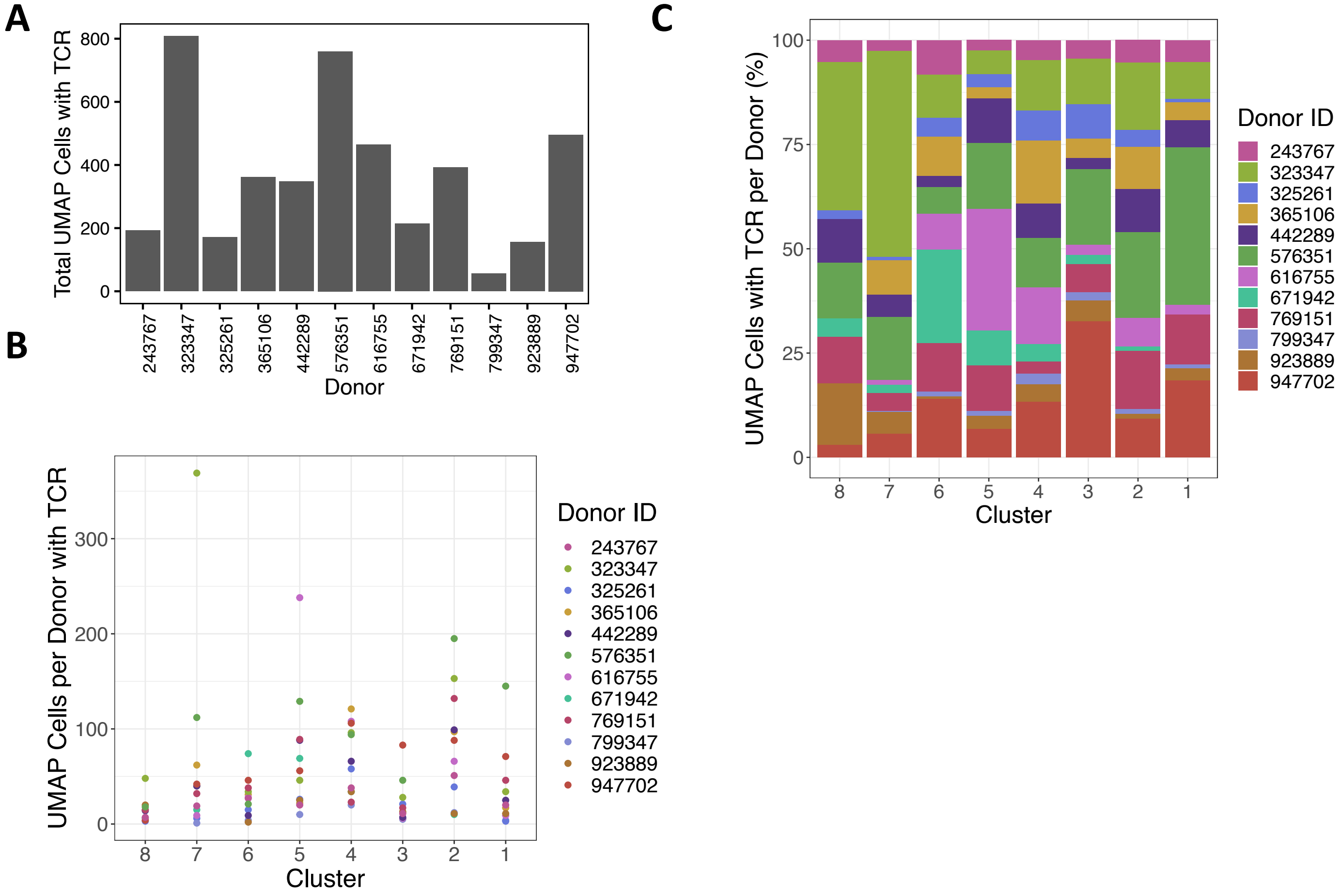

Supplementary Figure 14. TCR sequences were recovered for multiple donors per cluster, though total sequencing depth varied between donors. TCR statistics were analyzed after filtering the TCR data (see Methods) and matching cells with a retained TCR to cells that were retained following scRNA-seq filtering based on transcript depth and sequence quality. n = 12 donors sampled for dual scRNA-seq and TCR-seq, 6 R, 6 NR, 104 wks post-treatment. a) Total cells in each donor that have a sequenced *TRA-TRB* pair that come from cells retained after scRNA-seq QC. b) The total number of cells per cluster with a sequenced TCR *TRA-TRB* pair, plotted by color for each donor. c) The percentage of cells from each donor with a *TRA-TRB* per cluster.

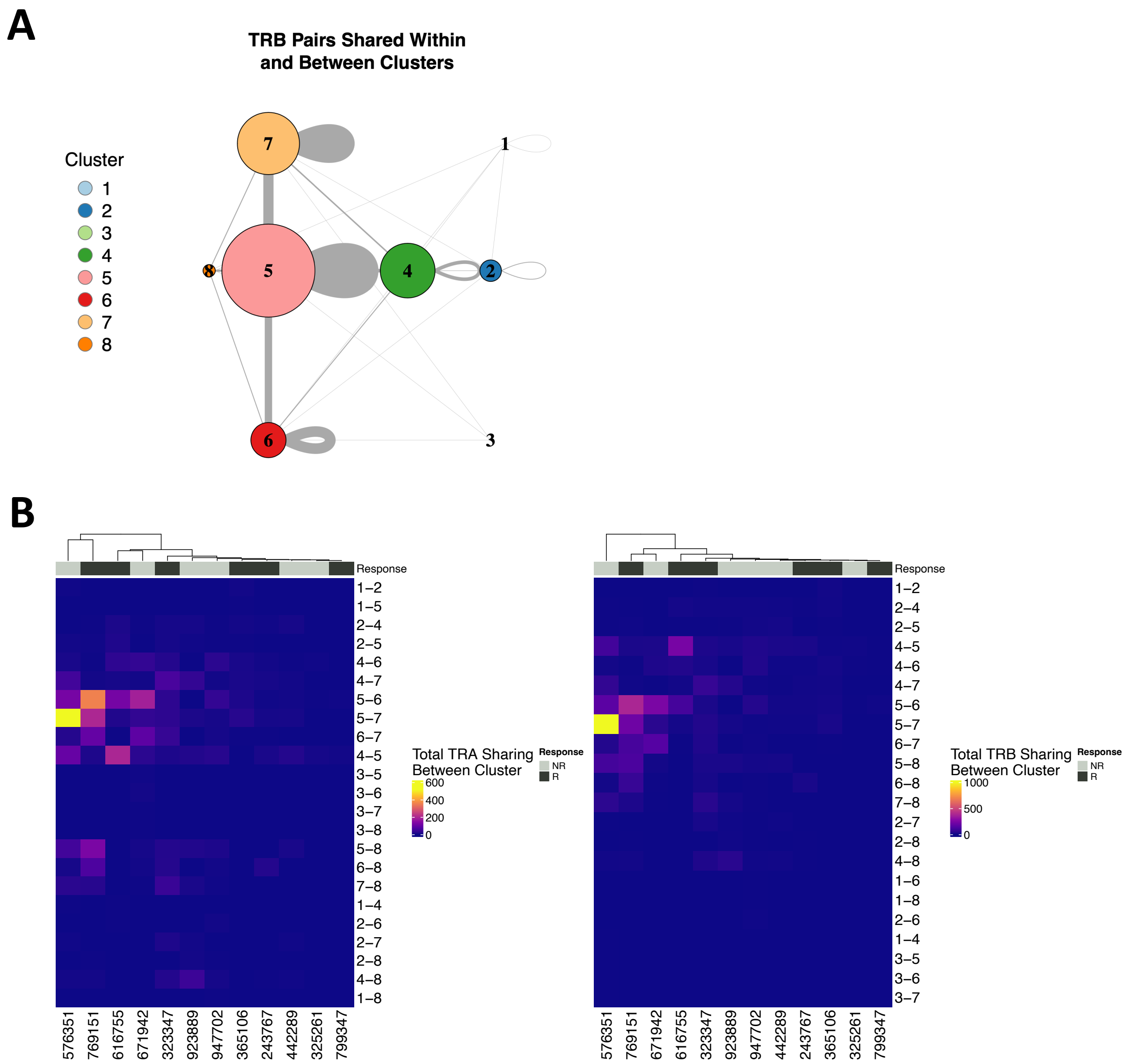

Supplementary Figure 15. TCR sharing between clusters reveals highest sharing between cluster 4 and exhausted clusters despite donor variability. a) Aggregated *TRB* chain sharing across combinations of clusters plotted as a network visualization with custom layout and color scheme in igraph to replicate scRNA-seq Leiden clustering and UMAP dimensionality reduction layout and color[1]. Nodes (circles) are sized by the total number of cells with a sequenced TCR per cluster. Straight edges (gray lines) indicate *TRB* sharing between clusters. Curved edges (gray loops) indicate *TRB* expansion within a cluster. Edge width is scaled to represent the level of *TRB* sharing. b) *TRA* (left) or *TRB* (right) sharing between cluster combinations per donor, colored by the total amount of sharing between cells. Rows are labelled by each combination of clusters that share a TCR (“1-2” indicates TCR sharing between clusters 1 and 2). N = 12 donors sampled for dual scRNA-seq and TCR-seq, 6 R, 6 NR, 104 wk post-treatment.

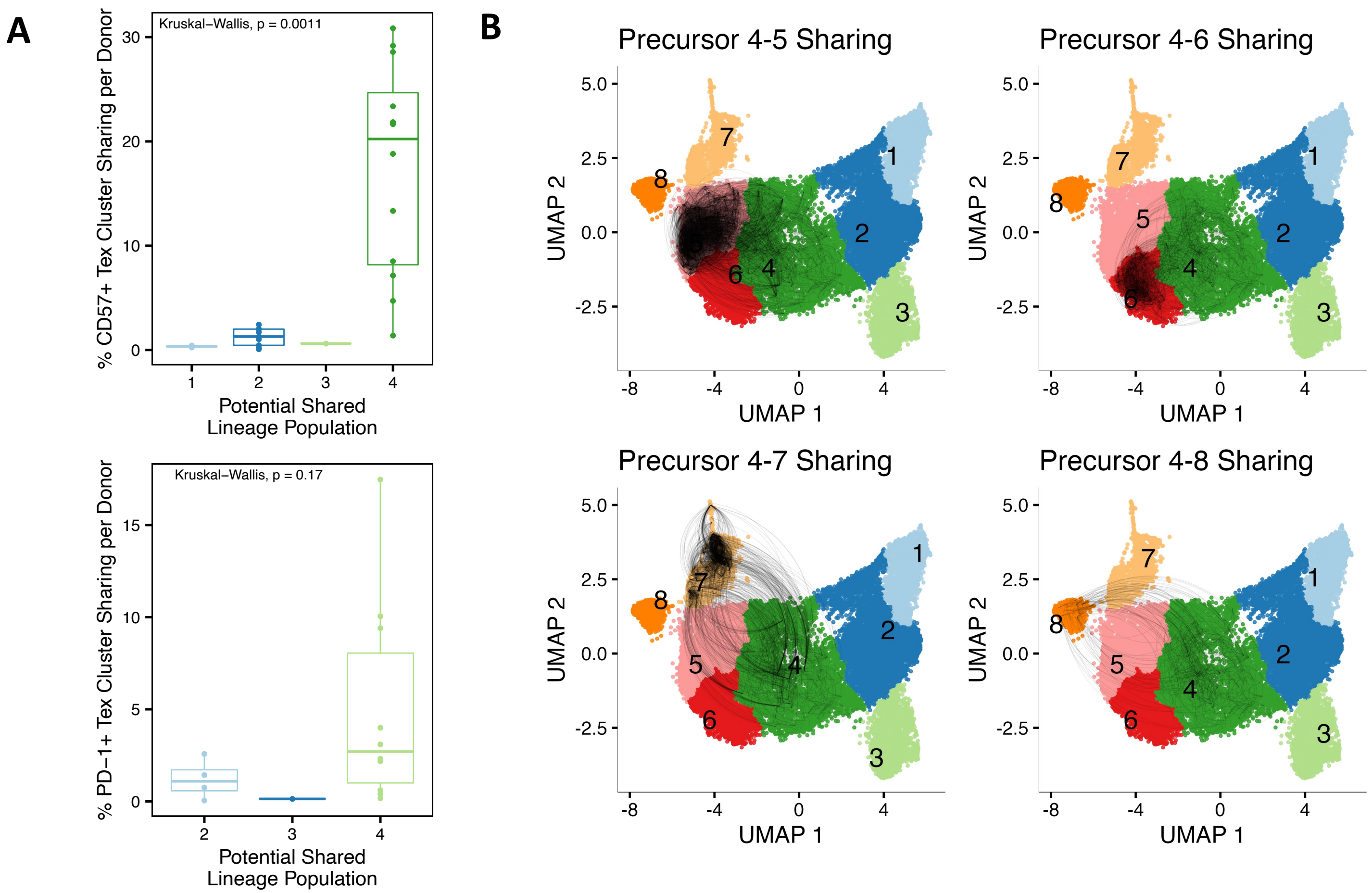

Supplementary Figure 16. Patterns of *TRA* sharing with CD57⁺ Tex and PD-1⁺ Tex clusters suggest cluster 4 as a shared precursor. a) The percentage of CD57⁺ Tex (top) or PD-1⁺ Tex (bottom) cells per donor that share a TCR with potential precursor clusters 2, 3, or 4. For both Tex populations, sharing is increased with cluster 4. This pattern is significant in the CD57⁺ Tex population (Kruskal-Wallis $p \leq 0.05$). Boxes extend from the first to the third quartiles (interquartile range) with a line in the middle that represents the median. Lines extending from the boxes (whiskers) represent variability outside the interquartile range. Population differences were determined using Kruskal–Wallis tests. b) UMAP dimensionality reduction plot depicting shared *TRA* chains between cluster 4 and each Tex cluster with curved segments.

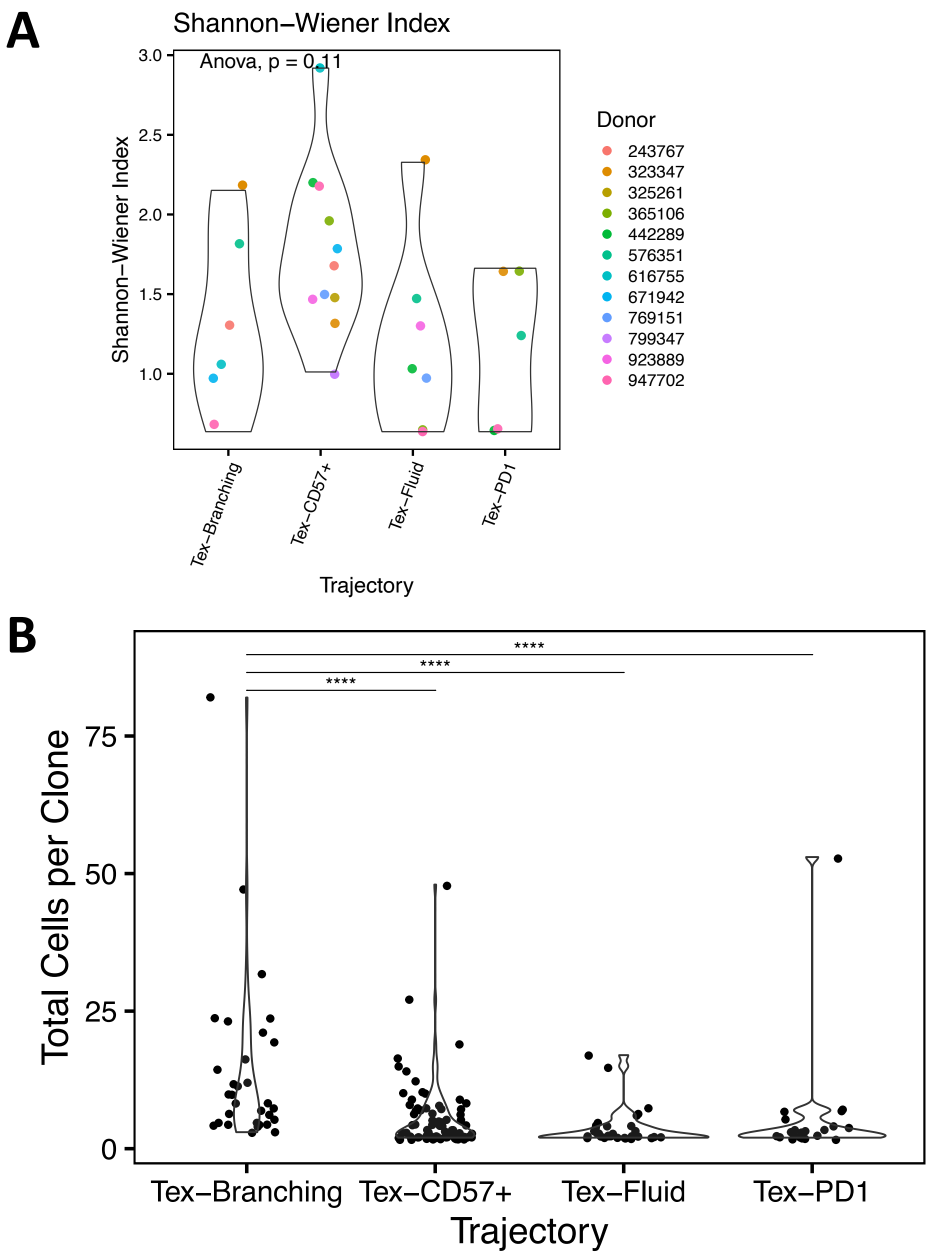

Supplementary Figure 17. Differentiation trajectories had similar *TRA* diversity, though Tex-Branching clones were most abundant across donors. a) The richness and evenness of individual *TRA* clones within a donor for each differentiation trajectory was calculated using the Shannon-Wiener Index. Differentiation trajectories did not differ in their overall diversity (One-way ANOVA, p-value = 0.11). The width of the violins represents the number of cases that have the values on the y-axis, and the shape shows the distribution of the data. b) The total number of clones possessing each *TRA* chain per differentiation trajectory across all donors were compared using the Pairwise Wilcoxon rank sum test (**** = adjusted p-value ≤ 1e-04). Tex-Branching clones were most abundant. n = 12 donors sampled for dual scRNA-seq and TCR-seq, 6 R, 6 NR, 104 wk post-treatment.

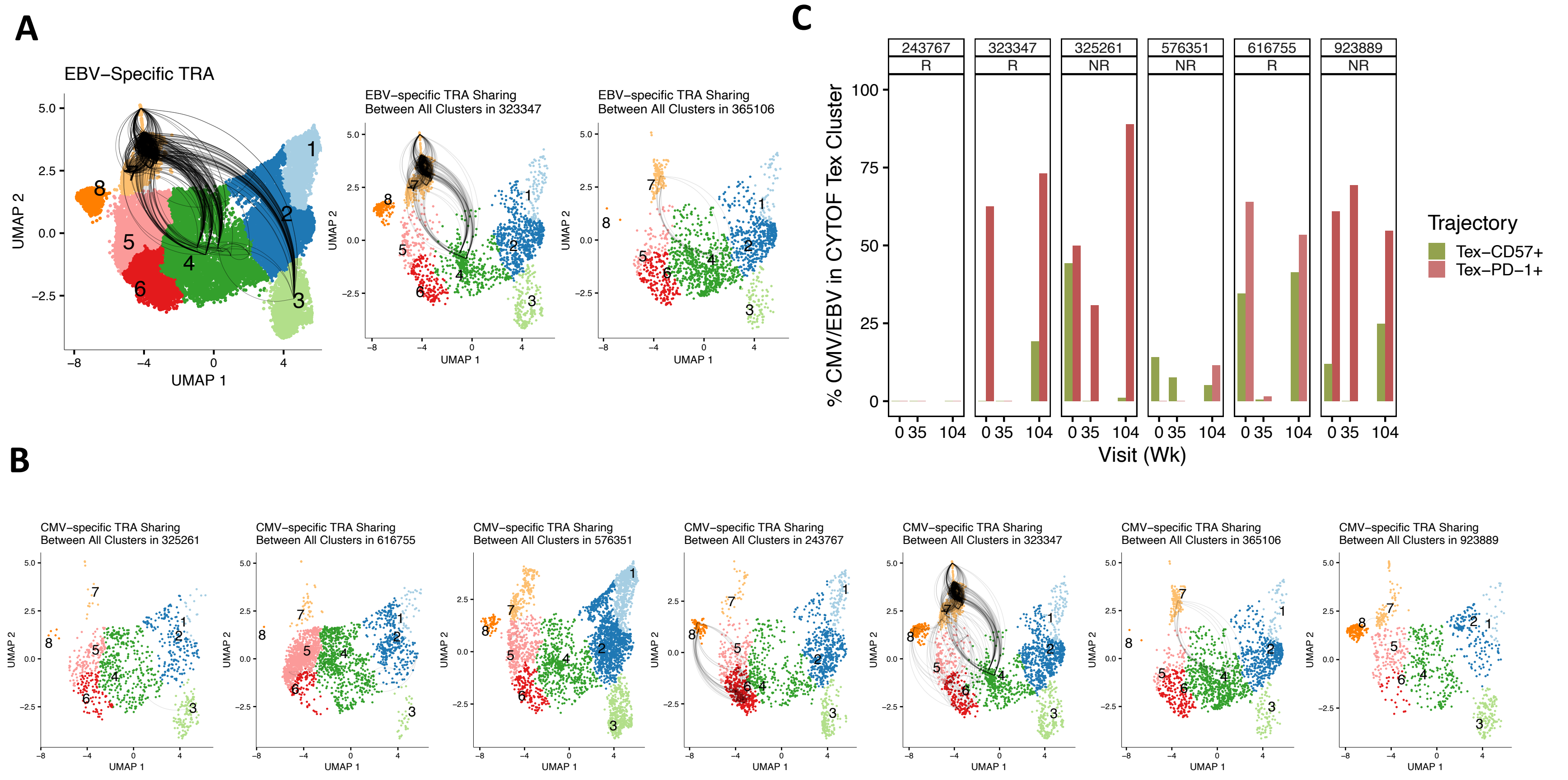

Supplementary Figure 18. CMV/EBV-specific *TRA*s are shared across multiple trajectories and are present in both PD-1$^+$ and CD57$^+$ Tex populations. a) UMAP dimensionality reduction connecting cells with the same EBV *TRA* specificity between or within clusters combined across all donors (left) or shown for only a subset of two donors (middle, right). EBV-specific cells were identified in the PD-1$^+$ Tex cluster 7 but not CD57$^+$ Tex clusters. N = 12 donors sampled for dual scRNA-seq and TCR-seq, 6 R, 6 NR, 104 wk post-treatment. b) UMAP dimensionality reduction connecting cells with the same CMV *TRA* specificity between or within clusters for the 7 individual donors with CMV-specific cells. CMV-specific cells were identified in both PD-1$^+$ Tex (cluster 7) and CD57$^+$ Tex clusters (cluster 5, 6, and 8). N = 12 donors were sampled for dual scRNA-seq and TCR-seq, 6 R, 6 NR, 104 wk post-treatment. c) The percent of CMV or EBV tetramer-specific TIGIT$^+$KLRG1$^+$ cells in either the PD-1$^+$ or CD57$^+$ Tex population identified by CYTOF at week 0, 35, or 104 using published procedures[13,14]. A subset of the 6 R donors sampled for dual scRNA-seq/TCR-seq data were sampled for CMV/EBV specificity in this CYTOF analysis. Multiple patients possessed antigen-specific cells with common CMV/EBV specificity in both PD-1$^+$ and CD57$^+$ Tex populations.

Supplementary References

1. Csardi, G. & Nepusz, T. The igraph software package for complex network research. *InterJournal, complex systems* **1695**, 1–9 (2006).
2. Dufort, M. J., Greenbaum, C. J., Speake, C. & Linsley, P. S. Cell type-specific immune phenotypes predict loss of insulin secretion in new-onset type 1 diabetes. *JCI Insight* **4**, (2019).
3. Li, J. *et al.* KIR(+)CD8(+) T cells suppress pathogenic T cells and are active in autoimmune diseases and COVID-19. *Science* **376**, eabi9591 (2022).
4. Daniel, B. *et al.* Divergent clonal differentiation trajectories of T cell exhaustion. *Nature Immunology* **23**, 1614–1627 (2022).
5. Zheng, L. *et al.* Pan-cancer single-cell landscape of tumor-infiltrating T cells. *Science* **374**, abe6474 (2021).
6. Daniel, B. *et al.* Divergent clonal differentiation trajectories of T cell exhaustion. *Nat Immunol* **23**, 1614–1627 (2022).
7. Long, S. A. *et al.* Partial exhaustion of CD8 T cells and clinical response to teplizumab in new-onset type 1 diabetes. *Sci Immunol* **1**, (2016).
8. Giles, J. R. *et al.* Human epigenetic and transcriptional T cell differentiation atlas for identifying functional T cell-specific enhancers. *Immunity* **55**, 557-574 e7 (2022).
9. Fridman, A. L. & Tainsky, M. A. Critical pathways in cellular senescence and immortalization revealed by gene expression profiling. *Oncogene* **27**, 5975–5987 (2008).
10. Purcell, M., Kruger, A. & Tainsky, M. A. Gene expression profiling of replicative and induced senescence. *Cell Cycle* **13**, 3927–3937 (2014).
11. Gillespie, M. *et al.* The reactome pathway knowledgebase 2022. *Nucleic Acids Res* **50**, D687–D692 (2022).

12. Saul, D. *et al.* A new gene set identifies senescent cells and predicts senescence-associated pathways across tissues. *Nat Commun* **13**, 4827 (2022).

13. Wiedeman, A. E. *et al.* Autoreactive CD8+ T cell exhaustion distinguishes subjects with slow type 1 diabetes progression. *J. Clin. Invest.* **130**, 480–490 (2020).

14. Diggins, K. E. *et al.* Exhausted-like CD8+ T cell phenotypes linked to C-peptide preservation in alefacept-treated T1D subjects. *JCI Insight* **6**, (2021).
