# Supplementary material for: Interconnected lineage trajectories link conventional and natural killer (NK)-like exhausted CD8+ T cells beneficial in type 1 diabetes
Source: Commun Biol. 2024 Jun 27;7:773. doi: 10.1038/s42003-024-06456-3 (PMC11211332; doi:10.1038/s42003-024-06456-3)
Supplement: Supplementary file 2 — Description of Additional Supplementary Files [file 42003_2024_6456_MOESM2_ESM.pdf]

Description of Additional Supplementary Files

File name: Supplementary Data 1

Description: Chromatin peaks significantly differentially accessible (DA) between PD-1+ Tex vs DN, CD57+ Tex vs DN, and CD57+ Tex vs PD-1+ Tex and annotated to a gene. All DA sites were significant at an FDR-adjusted p-value threshold £ 0.05. Sheet 1) sites increased in accessibility in CD57+ Tex vs DN. Sheet 2) sites increased in accessibility in PD-1+ Tex vs DN. Sheet 3) sites with increased accessibility in both CD57+ Tex vs DN and PD-1+ Tex vs DN. Sheet 4) sites with increased accessibility in CD57+ Tex vs DN (positive Fold values) and increased accessibility in PD-1+ Tex vs DN (negative Fold values).

File name: Supplementary Data 2

Description: Differential expression within CD57+ Tex scRNA-seq clusters. Sheets 1-3) Differentially expressed genes between individual pairwise comparisons of scRNA-seq CD57+ Tex clusters 5, 6, and 8. Sheet 4) Top specific markers for each scRNA-seq UMAP cluster as identified by Monocle.

File name: Supplementary Data 3

Description: Genes significantly differentially expressed between PD-1+ and CD57+ Tex scRNA-seq clusters determined by linear regression in Monocle. Sheet 1) Due to order of factors in analysis, negative log fold change (normalized effect) values indicate genes with increased differential expression in CD57+ Tex clusters 5, 6 and 8. Sheet 2) Positive log fold change (normalized effect) values indicate genes with increased differential expression in PD-1+ Tex cluster 7.

File name: Supplementary Data 4

Description: Genes significantly differentially expressed between alefacept Responders (R) and Non-Responders (NR) within each the PD-1+ and CD57+ Tex populations. For both comparisons, positive values indicate significantly increased expression in R, while negative values indicate significantly increased expression in NR. Sheet 1) R vs NR within all CD57+ Tex clusters (5 ,6, 8). Sheet 2) R vs NR in the PD-1+ Tex cluster 7.

File name: Supplementary Data 5

Description: De novo transcription factor motif enrichment within differentially accessible chromatin regions identified using HOMER. Sheet 1) De novo transcription factor (TF) motifs identified in differentially accessible chromatin peaks increased in CD57+ Tex vs CD57- (PD-1+ Tex). Only motifs with p-value £ 1e-12 were retained for analysis. Sheet 2) Matches of de novo identified TF motifs increased in CD57+ Tex vs CD57- (PD-1+ Tex) to known motifs. Sheet 3) De novo

transcription factor (TF) motifs identified in differentially accessible chromatin peaks decreased in CD57+ Tex vs CD57- (PD-1+ Tex). Only motifs with p-value £ 1e-12 were retained for analysis. Sheet 4) Matches of de novo identified TF motifs decreased in CD57+ Tex vs CD57- (PD-1+ Tex) to known motifs.

File name: Supplementary Data 6

Description: Differentially expressed genes between CD57+ Tex clusters (5, 6, and 8) and all other clusters. Sheet 1) All significantly differentially expressed genes (q-value £ 0.05) in CD57+ Tex clusters vs all other clusters as determined by Monocle linear regression.

File name: Supplementary Data 7

Description: Antibodies used for cytometry experiments.

File name: Supplementary Data 8

Description: The source data behind the graphs in the paper.
